# BioCNT-TLS engineered systemic mobile resistome for sustainable crop protection

**DOI:** 10.3389/fpls.2026.1903346

**Published:** 2026-08-24

**Authors:** Wenbo Li, Tahir Farooq, Muhammad Dilshad Hussain, Christopher A. Brosnan, Huanhuan Chen

**Affiliations:** 1 College of Biology and Food Engineering, Qujing Normal University, Qujing, China; 2 MARA-Key Laboratory of Surveillance and Management for Plant Quarantine Pests, College of Plant Protection, China Agricultural University, Beijing, China; 3 Centre for Horticultural Science, Queensland Alliance for Food and Agriculture Innovation, University of Queensland, Brisbane, QLD, Australia

**Keywords:** nanobionics, phloem-mediated transport, plant virus management, SIGS, sustainable crop protection, SWCNTs, systemic mobile resistome, tRNA-like structure

## Abstract

The global agricultural sector faces an existential threat from viral quasispecies that bypass traditional resistance through high-frequency mutation and rapid systemic colonization. Conventional strategies, including chemical pesticides and transgenic modifications, are increasingly constrained by environmental toxicity, regulatory hurdles, and the biological arms race of viral evasion. This review critically expands upon the transformative potential of biocompatible carbon nanotubes conjugated with tRNA-like structures as a non-transgenic, systemic delivery platform for RNA interference effectors. We analyze the mechanistic synergy between nanomaterial-mediated incorporation and vascular trafficking, proposing a systemic mobile resistome concept, in which BioCNT-TLS complexes may provide transgene-free, RNA-mediated interference distributed across aerial tissues from a single foliar application, an effect demonstrated in a single recent study and not yet validated at the whole-plant or field scale. By synthesizing data on RNAi pyramiding, spray-to-edit CRISPR technologies, and the ecotoxicological footprint of carbon nanomaterials, we provide a blueprint for the future of precision crop protection.

## Graphical Abstract

**Figure:**
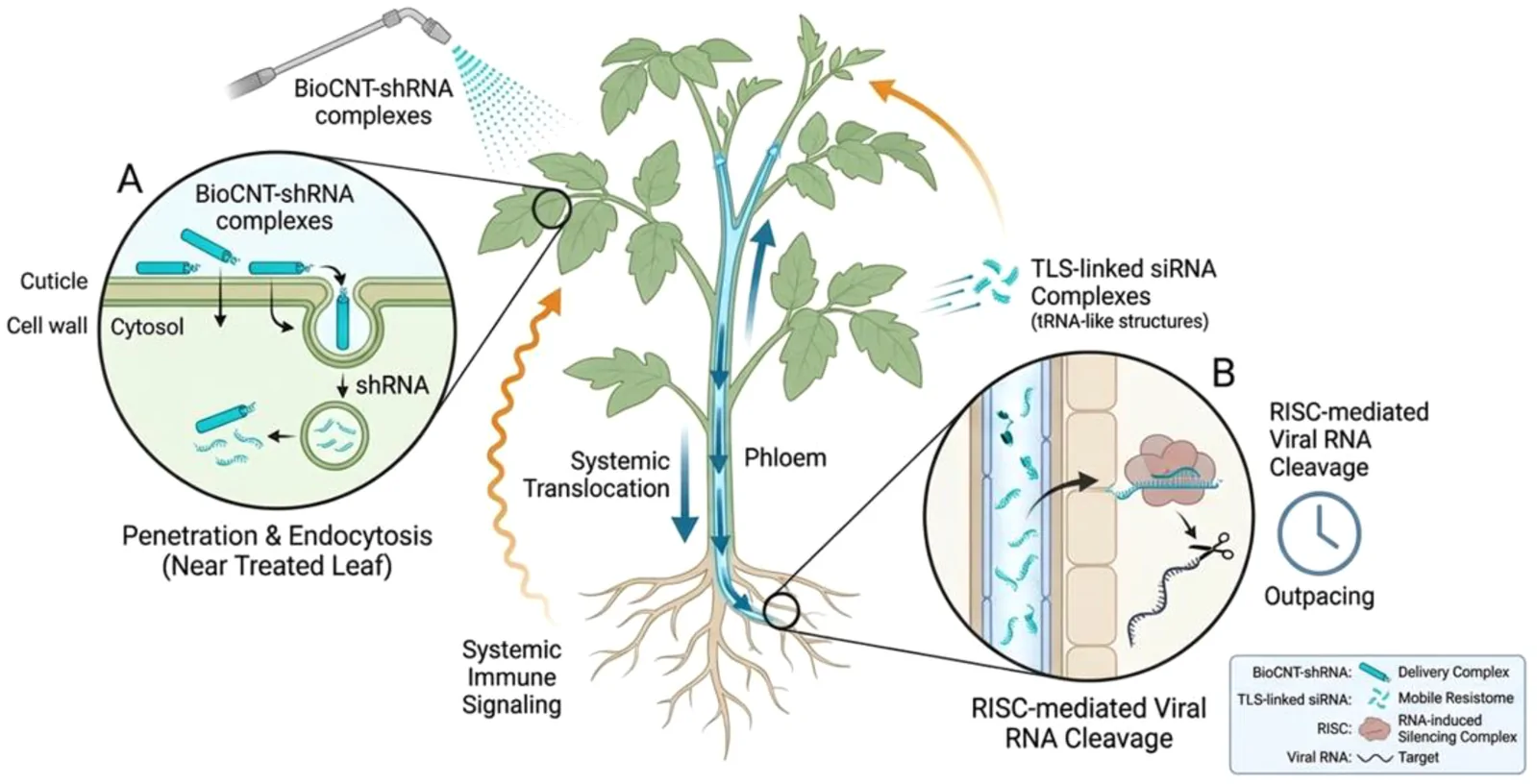
Engineered BioCNT-shRNA complexes enable rapid, systemic antiviral immunity in plants. **(A)** The complexes penetrate the leaf cuticle and cell wall via endocytosis, releasing shRNA into the cytosol. **(B)** The shRNA is processed into TLS-linked siRNAs, which undergo systemic translocation through the phloem to distant tissues. These mobile siRNAs are incorporated into the RNA-induced silencing complex (RISC) to mediate the cleavage of target viral RNA, outpacing viral replication and providing whole-plant protection.

## 1 Background: the biological impasse in plant virology

The intensification of global agriculture has inadvertently created a monoculture-heavy landscape that is highly susceptible to viral epidemics, leading to estimated annual losses exceeding $30 billion (Tatineni and Hein, 2023). The comparative suppression levels of these pathogens using nanobionic delivery are detailed in **Table 1**. Unlike fungal or bacterial pathogens, viruses are intracellular parasites that hijack the host’s molecular machinery, making direct chemical cures virtually non-existent (Ghosh et al., 2023). Current management strategies rely almost exclusively on the exclusion of insect vectors and the eradication of infected hosts, both of which are reactive and environmentally taxing (Rank and Koch, 2021; Xi et al., 2025). Furthermore, the rapid evolution of viral genomes allows for the quick emergence of resistant variants that render single-gene host resistance obsolete within a few growing seasons (Nicaise, 2014). The persistent reliance on vector control highlights a fundamental failure in our ability to engage with the viral pathogen at the genomic level. Therefore, we must develop proactive, sequence-specific molecular barriers that can be deployed with the same ease as traditional agrochemicals.

**Table 1:** Plant SIGS studies-foliar/topical dsRNA or sRNA.

Host (species, tissue)	Pathogen/target	Cargo (size)	Application	Suppression/protection outcome	Duration	Citation
*Nicotiana benthamiana*, intact leaf, abaxial side	*GFP* transgene	siRNA on SWNTs (size not specified)	Foliar spray (abaxial)	~70–80% siRNA-SWNT internalization in 6 h; GFP expression silenced for ~3 d; silencing abolished by day 7 (siRNA degradation); +12 h residence-time extension vs naked siRNA	3 d silencing peak	(Balusamy et al., 2023)
*Vigna unguiculata* (cowpea), true leaves	Pepper mild mottle virus (PMMoV; *Tobamovirus capsici*); Cucumber mosaic virus (CMV; Cucumovirus CMV)	LDH-BioClay dsRNA complex	Foliar spray (1:3 dsRNA: LDH)	dsRNA detectable on leaf surface ≥ 30 d; antiviral protection extended to 20 d post single-spray	20 d (vs ~5–7 d naked/*Escherichia coli* -extract)	(Balusamy et al., 2023)
*N. benthamiana*; *Arabidopsis*; *N. tabacum*	CMV, PMMoV, Bean common mosaic virus (BCMV; *Potyvirus phaseovulgaris*) (aphid-borne)	LDH-bound dsRNA	Foliar spray	Protection ≈ 20 d; window of antiviral activity ≈ 26 d in some settings	20–26 d	(Worrall et al., 2019)
*Cucumis sativus*	Cucumber green mottle mosaic virus (CGMMV; *Tobamovirus viridimaculae*)	dsRNA (bacterial expression; agroinfiltration)	Spraying	Virus resistance reported	not specified	(Tatineni and Hein, 2023)
*Cucumis sativus, Cucurbita pepo, Citrulus lanatus*	Zucchini yellow mosaic virus (ZYMV; *Potyvirus cucurbitaflavitesselati*)	dsRNA 588 bp *HC-Pro* + 498 bp CP; vsiRNAs (20 µL, 40–60 µg)	Co-inoculation by spraying + mechanical rubbing; naked delivery	ZYMV-derived vsiRNAs detected by stem-loop RT-PCR in local AND systemic leaves	Systemic spread confirmed	(Das and Sherif, 2020)
Pisum sativum	Pea seed-borne mosaic virus (PSbMV; Potyvirus pisumsemenportati)	dsRNA (in vitro synthesis)	Spraying	Virus resistance	not specified	(Tatineni and Hein, 2023)
*S. lycopersicum* (tomato)	CMV; Tomato leaf curl virus (ToLCV; *Begomovirus solanumaustraliaense*)	dsRNA (in vitro synthesis)	Mechanical inoculation	Virus resistance	not specified	(Tatineni and Hein, 2023)
*S. lycopersicum*	Tomato spotted wilt virus (TSWV; *Orthotospovirus tomatomaculae*)	dsRNA (in vitro synthesis)	Mechanical inoculation	Virus resistance	not specified	(Tatineni and Hein, 2023)
*Zea mays* (maize)	Sugarcane mosaic virus (SCMV; *Potyvirus sacchari*)	hpRNA (bacterially expressed crude extract)	Spraying	Virus resistance	not specified	(Tatineni and Hein, 2023)
*N. benthamiana*	*GFP* transgene	22-nt siRNA; high-pressure spray	Bud spray vs leaf spray; high-pressure vs wiping/infiltration/gene-gun	High-pressure spray was the most efficient delivery route; 22-nt siRNA delivered by bud spraying was more efficient than leaf-spray for inducing RNAi	Single application	(Das and Sherif, 2020)
*Arabidopsis thaliana*	*EGFP* and *NPTII* transgenes	dsRNA (naked)	Direct spreading (no high-pressure device)	Effective EGFP and NPTII suppression reported	not specified	(Das and Sherif, 2020)
*Hordeum vulgare* (barley); aphid *Sitobion avenae* on distal leaf	*Shp* aphid gene; CYP51 fungal gene	791 nt CYP3-dsRNA^ATTO488^; Shp-dsRNA	Foliar spray on local leaf; aphid feeding on distal leaf	~60% reduction in aphid Shp mRNA; fluorescent dsRNA detected in phloem sap by stylectomy; SIGS-derived siRNAs processed by plant RNAi machinery; transferred via phloem to roots and to distal leaves; root protection against Fusarium infection	> 48 h (spray → aphid-feeding → qRT-PCR)	(Biedenkopf et al., 2020)
Vigna unguiculata; N. tabacum	PMMoV; CMV	BioClay LDH-dsRNA; naked or LDH-loaded Nib-dsRNA 480 bp	Foliar spray 100 µg/1 mL or 250 ng dsRNA	Plants protected from aphid-mediated virus transmission	20 d	(Worrall et al., 2019)

BioClay, layered-double-hydroxide–bioclay (trade name); CMV 2b, Cucumber mosaic virus 2b silencing-suppressor; *CP*, coat protein; *CYP3*, cytochrome P450 family 3 (gene); *CYP51*, cytochrome P450 family 51 (sterol 14α-demethylase); d, day; dsRNA, double-stranded RNA; *EGFP*, enhanced green fluorescent protein; *GFP*, green fluorescent protein; *GUS*, β-glucuronidase (reporter gene); h, hour; *HC-Pro*, helper component–proteinase; hpRNA, hairpin RNA; LDH, layered double hydroxide; mRNA, messenger RNA; *NPTII*, neomycin phosphotransferase II; nt, nucleotide; SIGS, spray-induced gene silencing; siRNA, small interfering RNA; sRNA, small RNA; vsiRNA, virus-derived small interfering RNA.

**Figure 1:**
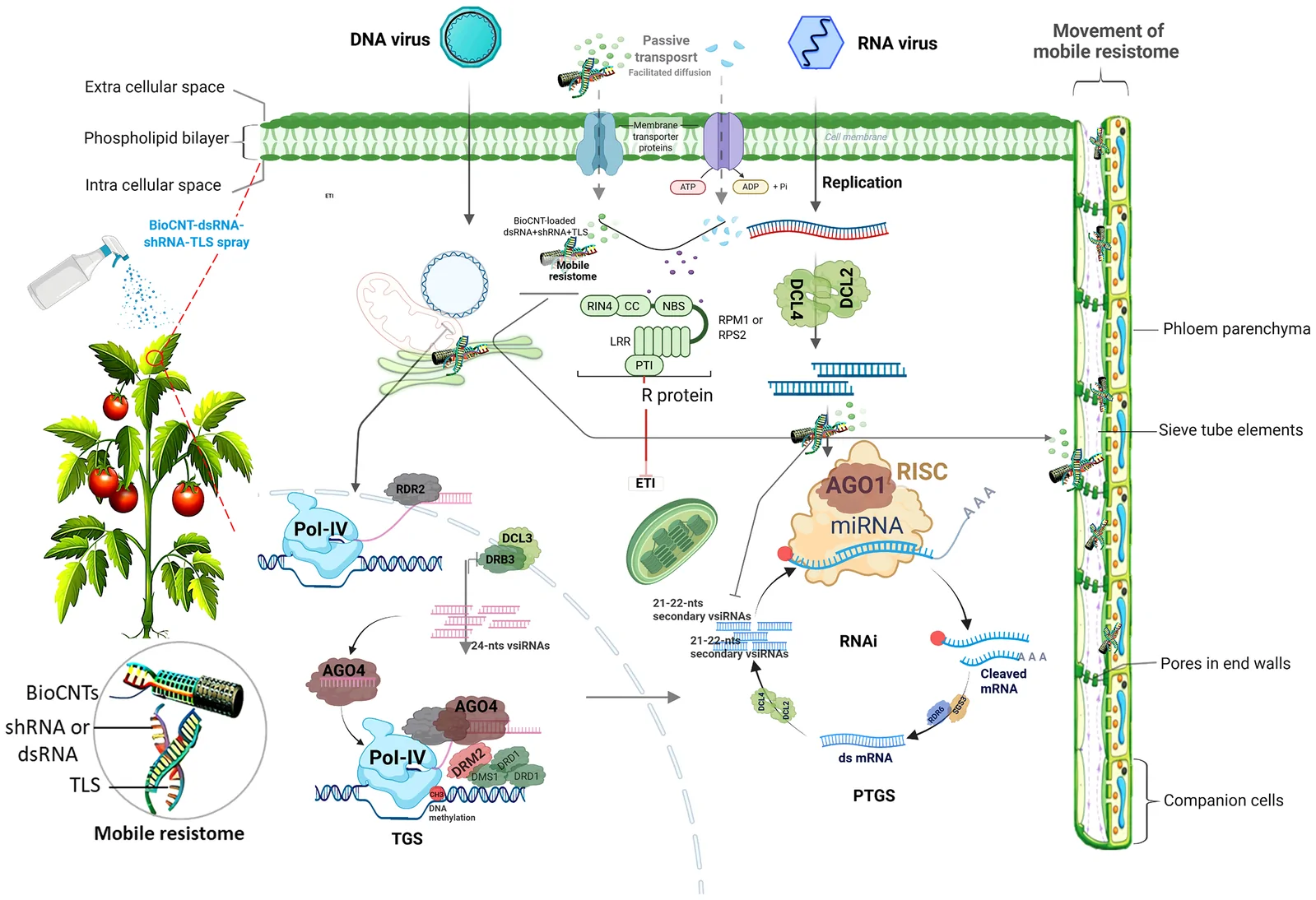
Engineering a phloem-mobile resistome for systemic antiviral defense. Induction of localized RNA interference (RNAi). Foliar-applied biological carbon nanotubes (BioCNTs; green rods) encapsulate and protect double-stranded RNA (dsRNA) or hairpin RNA (shRNA) effectors, facilitating their cytosolic delivery. Upon entry, the plant s endogenous RNAi machinery specifically DICER-like (DCL) proteins processes these effectors into small interfering RNAs (siRNAs). These siRNAs are subsequently loaded into the RNA-induced silencing complex (RISC), where Argonaute (AGO) proteins utilize them as guides to target and cleave complementary viral genomes, establishing localized resistance. Integration of tRNA-like structures (TLS) as molecular trafficking motifs enables the long-distance transport of the silencing signal. These motifs facilitate the loading of silencing determinants (likely as ribonucleoprotein complexes) into the phloem vasculature. This mechanism establishes a mobile resistome, whereby the silencing signal translocates from the site of application (source leaf) to distal, untreated tissues (e.g., sink leaves and roots). This systemic immunity preempts viral escape and provides durable, whole-plant protection.

RNA interference has long been heralded as the targeted intervention for viral management, offering a mechanism to silence specific viral genes through the induction of Dicer-like enzymes and the RNA-induced silencing complex (Hariharan et al., 2021). Indeed, virus-derived small interfering RNAs (vsiRNAs), generated via Dicer-like cleavage of viral dsRNA and loaded into Argonaute-containing RISC complexes, have been documented as the principal effector arm of antiviral RNAi in plant-virus systems (Arif et al., 2018), including members of the genus *Tenuivirus* infecting rice (Arif et al., 2017). However, the transition from lab-scale host-induced gene silencing to field-scale spray-induced gene silencing has been hindered by two critical bottlenecks: the instability of naked RNA molecules under UV and enzymatic pressure, and the lack of systemic movement from the site of application (Cagliari et al., 2019). Without a reliable delivery vehicle, the protective effects of spray-induced gene silencing (SIGS) are localized and transient, requiring frequent reapplications that are not economically viable (Dubrovina and Kiselev, 2019; Rank and Koch, 2021). The stability-mobility paradox is the primary hurdle preventing the commercialization of RNAi biopesticides. Solving this requires a multidisciplinary approach that integrates the structural resilience of nanotechnology with the biological intelligence of endogenous plant trafficking motifs (**Figure 1**).

Nanotechnology offers a promising path forward (Adeel et al., 2021a; Farooq et al., 2021; Farooq et al., 2024; Farooq et al., 2025), specifically through the use of single-walled carbon nanotubes that can be functionalized to carry genetic payloads (Demirer et al., 2020; Safdar et al., 2022). These biological carbon nanotubes (BioCNTs) leverage their high aspect ratio and needle-like geometry to bypass the formidable plant cell wall, protecting the RNA cargo from degradation while facilitating efficient cellular uptake (Husen and Siddiqi, 2014). The precise structural dimensions and loading parameters for these complexes are specified in **Table 2**. When integrated with tRNA-like structures that mimic the plant’s own systemic signaling molecules, these nanocarriers can navigate the phloem to establish a comprehensive, plant-wide immune state (Liu et al., 2026). The integration of BioCNTs and tRNA-like structures (TLS) motifs represents an engineered exploitation of the plant’s vascular system. However, the successful deployment of such a platform requires a deep understanding of the energy-dependent processes governing nanocarrier-membrane interactions and the regulatory pathways of the phloem source-to-sink transport.

**Table 2:** Physicochemical and architectural specifications of nanocarriers used in plant RNAi delivery.

Nanocarrier	Composition/dimensions	Surface/ζ-potential	Loading & N/P	Functional outcome in plants	Citation
SWNT	Single-walled carbon tube, D ≈ 1–2 nm, L 50 nm – 1 cm; tubular “nano-needle” morphology	Functionalized with PEI or PDDA (cationic polymer wrap) for electrostatic siRNA loading	Positively charged surface binds anionic siRNA; wrapping density not numerically specified in primary text	Efficient cellular uptake by mammalian cells; furnished the chemistry later adapted for plant RNAi (see Balusamy 2023 below)	(Draz et al., 2014)
SWNT–siRNA complex	SWNT with adsorbed siRNA	Cationic (PEI/PDDA-functionalized)	Cargo adsorbed by charge interaction (N/P not numerically specified in excerpts)	70–80% siRNA-SWNT internalization on abaxial *N. benthamiana* leaves after 6 h incubation; GFP silencing for ~3 d; +/- 12 h residence-time benefit over naked siRNA	(Balusamy et al., 2023)
LDH (layered double hydroxide) nanosheet — BioClay	Inorganic layered clay sheet, biocompatible; degrades under mildly acidic conditions (CO_2_ + humidity)	Surface chemistry: cation-exchangeable	dsRNA loaded at ~1:3 dsRNA: LDH ratio; released by acid-driven dissolution	30-day leaf-surface detectability of dsRNA; 20-day antiviral protection against PMMoV/CMV after single spray	(Tatineni and Hein, 2023)
Chitosan/dsRNA nanoparticle	Chitosan polysaccharide (crustacean-shell derived); biocompatible, non-toxic	Cationic polymer chain; electrostatic interaction with anionic dsRNA	Spontaneous nanocomplex; size not numerically specified in cited excerpt	60% reduction in endosomal accumulation of CypHer-5E-labelled dsGFP in Sf9 cells vs naked dsRNA; supports endosomal escape; chitosan/dsIAP fed to *Spodoptera frugiperda* larvae → 47% mortality vs 25% with naked dsIAP; *Anopheles gambiae* chitosan/dsRNA against chitin-synthase transcripts → 62.8% mRNA reduction	(Pugsley et al., 2021)
Cationic cell-penetrating peptide — fusogenic HA + DRBD	Ribonucleoprotein particle: CPP–DRBD/dsRNA	Cationic CPP-DRBD surface	dsRNA bound via DRBD; complexes enhance stability at pH 5.5	80% *Anthonomus grandis Ag-ChSII* transcript reduction vs 30% for naked dsRNA; CPP-DRBD enhanced cellular uptake of Cy3-labelled dsRNA into Sf21 cells	(Pugsley et al., 2021)
Cationic polymer NP, Tobacco BY-2 protoplasts	CPN platform summarized in review	Cationic	not numerically specified in cited excerpt	> 76% transcript reduction of NtCesA-1 in Tobacco BY-2 protoplasts	(Pugsley et al., 2021)
PAMAM dendrimer (with fluorescent PDI core)	Polyamidoamine dendriplex; sub-100 nm	High positive charge; high water solubility	Loading by electrostatic complexation	*Bombyx mori* cells: knockdown at higher PAMAM concentrations; *Drosophila* S2 cells: 95.4% hemocytin transcript reduction; fluorescein-PAMAM/*Drosophila* decapentaplegic larvae body length reduced by 35%	(Pugsley et al., 2021)
PAMAM-dendrimer × dsRNA, *Arabidopsis thaliana*	Cationic PAMAM dendriplex	Cationic	not numerically specified in cited excerpt	STM mRNA reduced 84%; WER mRNA reduced 87%	(Pugsley et al., 2021)
Dendrimer-coated carbon nanotube	PAMAM-coated CNT	Cationic surface	dsRNA bound to dendrimer	*Tribolium castaneum*: 4th-instar larvae injected with PAMAM-CNT-dsRNAs showed significantly greater α-tub/mtpol transcript reduction after 72 h vs naked dsRNA control	(Pugsley et al., 2021)
Guanidinium-functionalized polymer	Cationic polymer	Cationic	not numerically specified in cited excerpt	> 80% *sfV-ATPase* knockdown in *Spodoptera frugiperda*	(Pugsley et al., 2021)
Star polycation — transdermal	Cationic star-shaped polymer	Cationic	not numerically specified in cited excerpt	Confirmed gene silencing in Aphis gossypii by transdermal delivery; sensitivity of RNA aphid sprays demonstrated	(Rank and Koch, 2021)
NPs in general	r = 1-dim hollow tube; D ≈ 1–2 nm, L 50 nm – 1 cm	“Large surface area; ultra-high functionalization; high penetration; ultimate electrical, thermal, mechanical strength”	High loading capacity	Non-biodegradable; unresolved toxic properties - flagged as disadvantage for translational use	(Draz et al., 2014)

BY-2, Tobacco Bright Yellow-2 (cell line); CPP, cell-penetrating peptide; CPN, cationic polymer nanoparticle; Cy3, cyanine 3 (fluorescent dye); CypHer-5E, proprietary pH-sensitive fluorophore; D, diameter; DRBD, double-stranded RNA-binding domain; ds*IAP*, double-stranded inhibitor-of-apoptosis protein; HA, hemagglutinin; L, length; NP, nanoparticle; N/P, nitrogen-to-phosphate (charge ratio); PDDA, poly (diallyldimethylammonium chloride); PEI, polyethyleneimine; RNP, ribonucleoprotein; Sf9, *Spodoptera frugiperda* cell line 9; Sf21, *Spodoptera frugiperda* cell line 21; siRNA, small interfering RNA; SWNT, single-walled carbon nanotube; ζ-potential, zeta potential.

## 2 The BioCNT architecture: physicochemical engineering for intact cells

**Figure 2:**
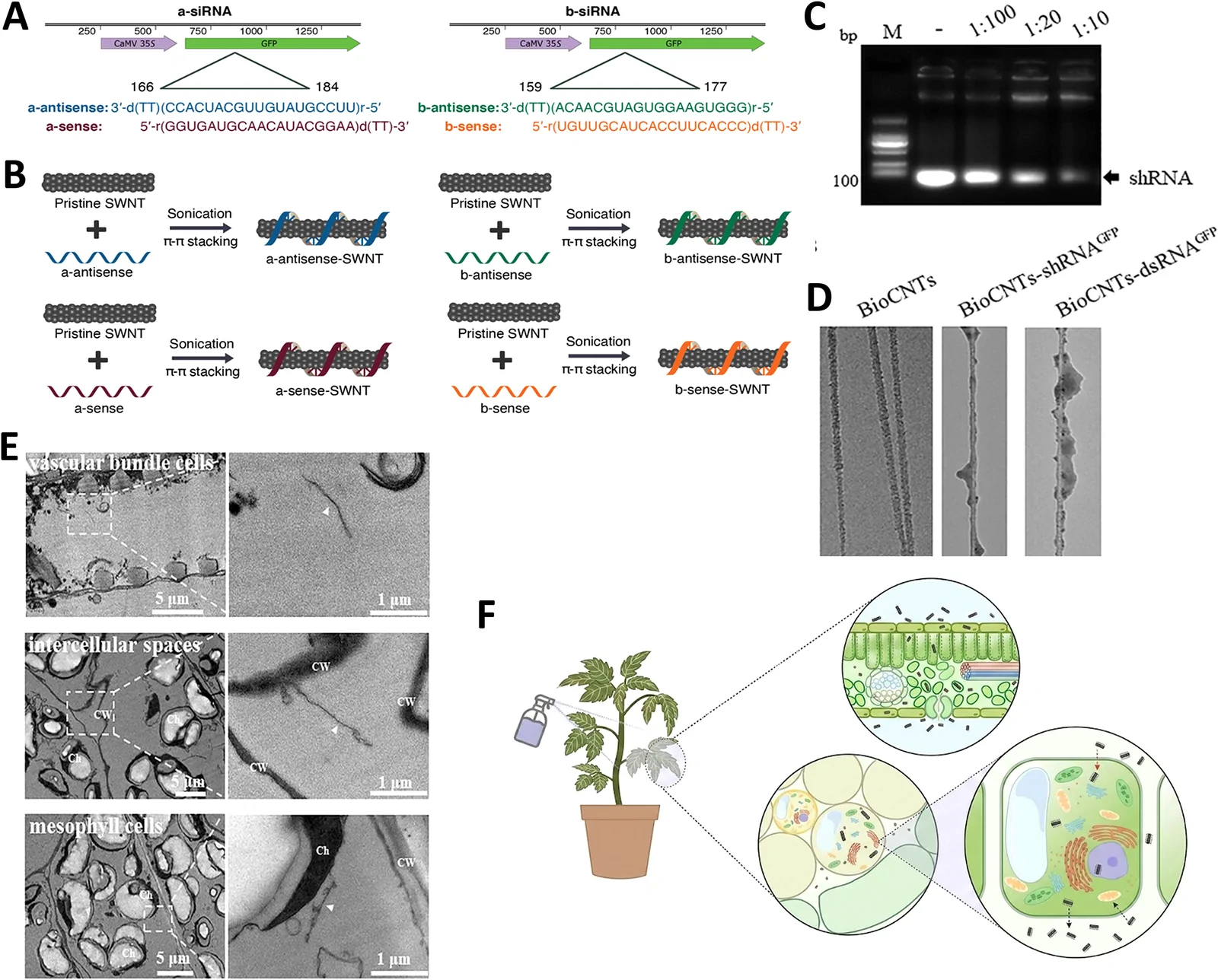
Synthesis, characterization, and cellular uptake of BioCNTs-shRNA. **(A)** Schematic of siRNA sequences targeting the *mGFP5* gene in *N. benthamiana*, showing original (a-siRNA) and *de novo* (b-siRNA) designs (Demirer et al., 2020). **(B)** Sonication-mediated loading of ssRNA onto single-walled carbon nanotubes via stacking interactions (Liu et al., 2026). **(C)** Agarose gel electrophoresis showing optimal shRNA loading at a 1:10 mass ratio (Liu et al., 2026). **(D)** TEM images of pristine BioCNTs, BioCNTs-shRNA, and BioCNTs-dsRNA (Liu et al., 2026). **(E)** TEM characterization of BioCNT-dsRNA entry into tomato leaf cells, with nanoparticles (arrowheads) visible in vascular bundles and mesophyll cytoplasm. **(F)** Schematic of BioCNT distribution in tomato leaves following foliar application, illustrating epidermal penetration and cytosolic accumulation (Liu et al., 2026).

The engineering of BioCNTs begins with the functionalization of single-walled carbon nanotubes to ensure biocompatibility and high cargo-loading efficiency. The values reported in **Tables 1**–**3** below represent a composite of (a) directly measured parameters from individual experimental studies, which are cited at the level of the relevant table cell, and (b) conceptual design parameters derived from synthesis chemistry. Each row is annotated accordingly (Mat Jalaluddin et al., 2023; Sede et al., 2026) (**Figure 2**). Pristine nanotubes are inherently hydrophobic and tend to aggregate, and are therefore typically wrapped in positively charged polymers such as polyethyleneimine or single-stranded DNA (ssDNA), which provides the electrostatic attraction necessary to bind negatively charged double-stranded RNA (dsRNA) or short hairpin RNA (shRNA). The optimization of carrier dose, particle size, and ζ-potential for symplastic delivery and antiviral efficacy is discussed comprehensively in the recent perspective (Sede et al., 2026), which provides a critical analysis of how these parameters constrain RNA bioavailability in planta. The polyethylenimine (PEI) wrapping strategy used here, and in the foundational experimental demonstrations of SWNT-mediated siRNA delivery to intact *Nicotiana benthamiana* leaf tissue, was chosen for its proven delivery efficiency rather than its intrinsic biocompatibility: Demirer et al. verified gene knockdown at the protein level and at the mRNA level, establishing the dose–response baseline against which all subsequent BioCNT-TLS designs are benchmarked (Demirer et al., 2020). The nitrogen-to-phosphate (N/P) ratio is a critical parameter; an excess of cationic charge can disrupt plasma membrane integrity, leading to localized cell death that counteracts the goal of systemic protection. Optimising electrostatic RNA adsorption on functionalised SWCNTs requires balancing carrier dose, particle size and ζ-potential; Sede et al. (2026) provide a critical, up-to-date analysis of how these parameters constrain symplastic delivery.

**Table 3:** Comparative performance of nanocarrier SIGS platforms.

Nanocarrier class	Host (species, tissue)	Pathogen/target	Cargo	Application	Suppression/protection outcome	Functional window	Provenance/stage	Primary reference (single, full)
SWNT (carbon nanotube)	*N. benthamiana*, intact leaf, abaxial	*GFP transgene*	siRNA	Foliar (abaxial touch)	70–80% cargo internalization in 6 h; ~3-d GFP silencing peak	3 d peak; lost by d 7	single-condition, abaxial touch	(Balusamy et al., 2023)
LDH	*Vigna unguiculata* true leaves; *N. benthamiana*/*N. tabacum*; *Arabidopsis*; *Citrus maxima* (vascular delivery)	Pepper mild mottle virus (PMMoV; *Tobamovirus capsici*); Cucumber mosaic virus (CMV; *Cucumovirus CMV*); Bean common mosaic virus (BCMV; *Potyvirus phaseovulgaris*; *Panonychus citri*; *Diaphorina citri*; *Aphis gossypii* (stylet feeders)	dsRNA 977 bp PMMoV; 330 bp CMV2b; 504 bp GUS; mite/aphid essential-gene dsRNA	Foliar spray (1:3 dsRNA: LDH); seed treatment; vascular delivery (stem uptake)	dsRNA detectable on leaf surface ≥ 30 d; first demonstration of sustained release; antisense activity expanded ~5–7 d → ~20 d and up to 26 d in some virus–host pairs	20–26 d	lab + greenhouse; not replicated in open field	(Tatineni and Hein, 2023)
Naked dsRNA (historical baseline)	*N. benthamiana, S. lycopersicum, cucurbits, V. unguiculata*	CMV; Tobacco mosaic virus (TMV; *Tobamovirus tabaci*); PMMoV; Zucchini yellow mosaic virus (ZYMV; *Potyvirus cucurbitaflavitesselati*); Tomato spotted wilt virus (TSWV; *Orthotospovirus tomatomaculae*); Pea seed-borne mosaic virus (PSbMV; *Potyvirus pisumsemenportati*); Tomato leaf curl virus (ToLCV; *Begomovirus solanumaustraliaense*); Sugarcane mosaic virus (SCMV; *Potyvirus sacchari*); Cucumber green mottle mosaic virus (CGMMV; *Tobamovirus viridimaculae*)	dsRNA (in vitro transcription or bacterial expression); hpRNA; ssRNA	Mechanical inoculation, spraying, high-pressure spraying	Functional antiviral resistance documented across 9+ virus–host pairs	5–7 d	lab-confirmed; field rarely replicated	(Tatineni and Hein, 2023)
High-pressure spray of dsRNA/siRNA (no carrier)	*N. benthamiana*	*GFP* transgene	dsRNA; 22-nt siRNA	High-pressure spray onto leaves/buds	Local AND systemic silencing of GFP; high-pressure > wiping/infiltration/gene-gun; bud spray > leaf spray for 22-nt siRNA	Single application	–	(Das and Sherif, 2020)
Escherichia coli -expressed crude dsRNA extract	*N. benthamiana*	PMMoV; Aalfalfa mosaic virus (AMV; *Alfamovirus AMV*); Tobacco etch virus (TEV; *Potyvirus nicotianainsculpentis*)	Crude HT115 E. coli lysate dsRNA	Foliar (co-inoculation)	Antiviral protection similar to purified dsRNA	5–7 d	lab; purification required for regulatory acceptance	(Fletcher et al., 2020)
Chitosan/dsRNA	*S. frugiperda* 3rd-instar larvae; *A. gambiae* larval aquatic stages; *Anopheles, Aedes*	*IAP*; chitin synthase	dsRNA	Oral feeding	47% mortality vs 25% naked; 62.8% chitin-synthase mRNA reduction	5 d post-feeding	lab	(Pugsley et al., 2021)
PAMAM dendriplex	*Drosophila* S2 cells; B. mori cells; Drosophila larvae; *T. castaneum*; *Arabidopsis thaliana*; *Ostrinia furnacalis*	*Hemocytin*, *TCTP*, decapentaplegic, *α-tub/mtpol*, *STM/WER, serpin-3, CHT10*	dsRNA	Cell transfection; oral feeding; injection	95.4% *hemocytin* reduction (S2); STM 84%, WER 87% reduction (*Arabidopsis*); CHT10 feeding → stunted growth, failure to moult; 51% *serpin-3* mRNA + undetectable protein	72 h post-injection	lab	(Pugsley et al., 2021)
Star polycation	*Aphis gossypii*	RNAi targets (transdermal)	dsRNA/siRNA	Transdermal on aphid cuticle	Gene-silencing confirmed in cotton aphid; species-specific formulation	single application	lab	(Rank and Koch, 2021)
CPP-DRBD/dsRNA (ribonucleoprotein particle)	Sf21 cells; *Anthonomus grandis*	*Ag-ChSII*; Cy3-dsRNA	HA-fusogenic CPP fused to DRBD + dsRNA	Cell uptake; oral feeding	80% Ag-ChSII transcript reduction; enhanced pH 5.5 stability	not specified	ab	(Pugsley et al., 2021)
E. coli HT115 lysate (plant-passage-free dsRNA)	*Vigna unguiculata*; chestnut, sweet orange, *Zea mays*, rice, *Brassica oleracea, Solanum tuberosum*	Plant viruses; coleopteran beetles; cockroaches; aphids; whiteflies; lepidopteran larvae	dsRNA (lysate or purified)	Spray, root drench, trunk injection, irrigation, seed coat	Coleopterans most susceptible; lepidopterans/hemipterans least; aphid uptake limited without carrier	5–13 d	lab + simulated field	(Rank and Koch, 2021)
Biedenkopf 2020 barley SIGS (no carrier for dsRNA uptake)	*Hordeum vulgare*; *Sitobion avenae* aphid on distal leaf	*Shp* (aphid); *CYP51*	791 nt CYP3-dsRNA_ATTO488_; Shp-dsRNA	Foliar spray; aphid stylectomy in distal segments	60% aphid Shp knockdown; SIGS-derived siRNAs detected in phloem sap; root dsRNA uptake demonstrated	> 48 h from spray to signal	lab	Biedenkopf D, Will T, Knauer T, et al., 2020. Systemic spreading of exogenous applied RNA biopesticides in the crop plant Hordeum vulgare. Environmental Microbiome 15:12. doi:10.1186/s41544-020-00052-3

*α-tub*, alpha-tubulin; *Ag-ChSII*, *Anthonomus grandis* chitin synthase II; *CHT10*, chitinase 10; CPP, cell-penetrating peptide; decapentaplegic, *Drosophila* decapentaplegic (dpp) orthologue; *hemocytin*, *Drosophila hemocytin* (immune gene); *mtpol*, mitochondrial DNA polymerase; NtCesA-1, *Nicotiana tabacum* cellulose synthase A catalytic subunit 1; PAMAM, polyamidoamine (dendrimer); *STM*, SHOOTMERISTEMLESS (transcription factor); *TCTP*, translationally controlled tumor protein; *WER*, WEREWOLF.

## 3 tRNA-like structures and the systemic mobile resistome

The defining feature of the mobile resistome is its ability to utilize the plant’s vascular system for long-distance transport. In natural systems, the plant uses specific RNA motifs, such as tRNA-like structures, to mark certain mRNAs for systemic movement via the phloem (Limera et al., 2017; Liu et al., 2026). These motifs are recognized by specialized RNA-binding proteins that facilitate their loading into the sieve elements (Das and Sherif, 2020; Brosnan et al., 2026). By incorporating these TLS sequences into synthetic shRNA constructs, researchers can trick the plant into transporting the antiviral signal from the sprayed source leaf to unsprayed sink tissues like young leaves, roots, and flowers (Liu et al., 2026). The use of TLS motifs as molecular zip codes is a pinnacle of bio-inspired engineering. However, the efficacy of this transport is highly dependent on the plant’s physiological state; environmental factors such as light intensity and water stress, which dictate phloem flow, may significantly alter the distribution and concentration of the systemic RNAi signal.

In a recent study using *N. benthamiana*, distal shRNA accumulation above the silencing threshold was reported at ~14 days post-application, with a 50-60% reduction in the target transcript levels in systemic leaves, consistent with pre-armed interception of incoming viral RNA (Biedenkopf et al., 2020; Liu et al., 2026). Whether these kinetics generalize to other host species, viral targets, or field conditions remains untested. Notably, the 14-day delay in reaching peak distal concentration represents a significant vulnerability window in the event of rapid viral outbreaks. Thus, researchers must investigate whether the addition of specific accelerator molecules, or the use of multiple trafficking motifs, can shorten this lead time to provide more immediate protection to new growth.

## 4 Pyramiding and the management of viral evolution

Viral evasion is a constant threat in agricultural pathology, driven by the emergence of quasispecies -highly diverse viral populations that can quickly adapt to selective pressures (Tatineni and Hein, 2023). To ensure the durability of the RNAi-mediated defense, a pyramiding strategy is employed, wherein multiple shRNAs are delivered simultaneously, each targeting a different, functionally immutable region of the viral genome (Nicaise, 2014). Typical targets include the replicase domains, which are essential for viral multiplication and generally show high sequence conservation (Wilson, 1993). Particularly, pyramiding is not merely a quantitative increase in protection but a qualitative shift in the evolutionary pressure applied to the pathogen. By forcing the virus to undergo multiple simultaneous mutations to survive, we effectively close the evolutionary exit and significantly extend the lifespan of the biopesticide.

If the kinetics reported by Liu et al., are taken as an upper boundary, an order of magnitude estimate can be derived assuming first order systemic transport. However, no validated mathematical model of BioCNT-TLS phloem loading and unloading currently exists. Also, the numerical values should be treated as illustrative rather than predictive (Nicaise, 2014). This evolution-resilient framework has been successfully tested against co-infections of DNA and RNA viruses, such as Tomato mosaic virus (ToMV; *Tobamovirus tomatotessellati*) and Tomato yellow leaf curl virus (TYLCV; *Begomovirus coheni*). The use of shRNAs, which are more compact and easily processed than long dsRNAs, allows for higher loading density on the BioCNT surface, facilitating the delivery of these complex, multi-target pyramids in a single dose (Liu et al., 2026). In this context, while pyramiding provides robust defense, the metabolic cost of maintaining a multi-target silencing response in the plant must be evaluated. If the plant diverts too much energy toward processing these exogenous RNAs, there may be a growth-defense trade-off that could impact crop yield in the absence of a viral challenge.

## 5 The nanoprimer strategy: optimizing delivery kinetics

Hypothetically, an extension of the mammalian nanoprimer concept (Saunders et al., 2020) -where nanocarriers pre-arm innate immune pathways-could in principle be adapted to plants. However, no direct experimental evidence currently demonstrates nanoprimer-type activity in plant systems; the discussion that follows should therefore be read as a conceptual framework for future investigation. Given that plant cells lack a reticuloendothelial-style clearance system; extracellular dsRNA and shRNA are subject to apoplastic RNase-mediated degradation, movement through the symplastic continuum via plasmodesmata, and sequestration within endosomal compartments. By applying a priming dose of non-functional nanocarriers prior to the therapeutic BioCNT-RNA dose, researchers can occupy the plant’s non-specific binding sites and extracellular nucleases, ensuring that a higher percentage of the active cargo reaches the phloem (Saunders et al., 2020; Zhu et al., 2022).

### 5.1 Overcoming the apoplastic barrier

The apoplast, consisting of the cell wall and the intercellular spaces, serves as a significant physical and chemical barrier to RNAi (Dubrovina and Kiselev, 2019; Brosnan et al., 2026). Mobile dsRNAs are often detected in the apoplast, where they can be intercepted by fungal pathogens but are also subject to degradation by secreted plant enzymes (Biedenkopf et al., 2020; Brosnan et al., 2026). BioCNTs facilitate the symplastic entry of these RNAs, bypassing the apoplastic trap and ensuring that the silencing signal is localized within the cytoplasm, where the RISC complex is active (Dubrovina and Kiselev, 2019; Zhu et al., 2022). Markedly, while the distinction between apoplastic and symplastic transport is the difference between transient and durable protection; by forcing the RNA into the symplast via BioCNTs, we transition from a surface-level shield to an internalized, cellular-level immune system.

## 6 Molecular interfacial engineering: designing the molecular velcro

**Figure 3:**
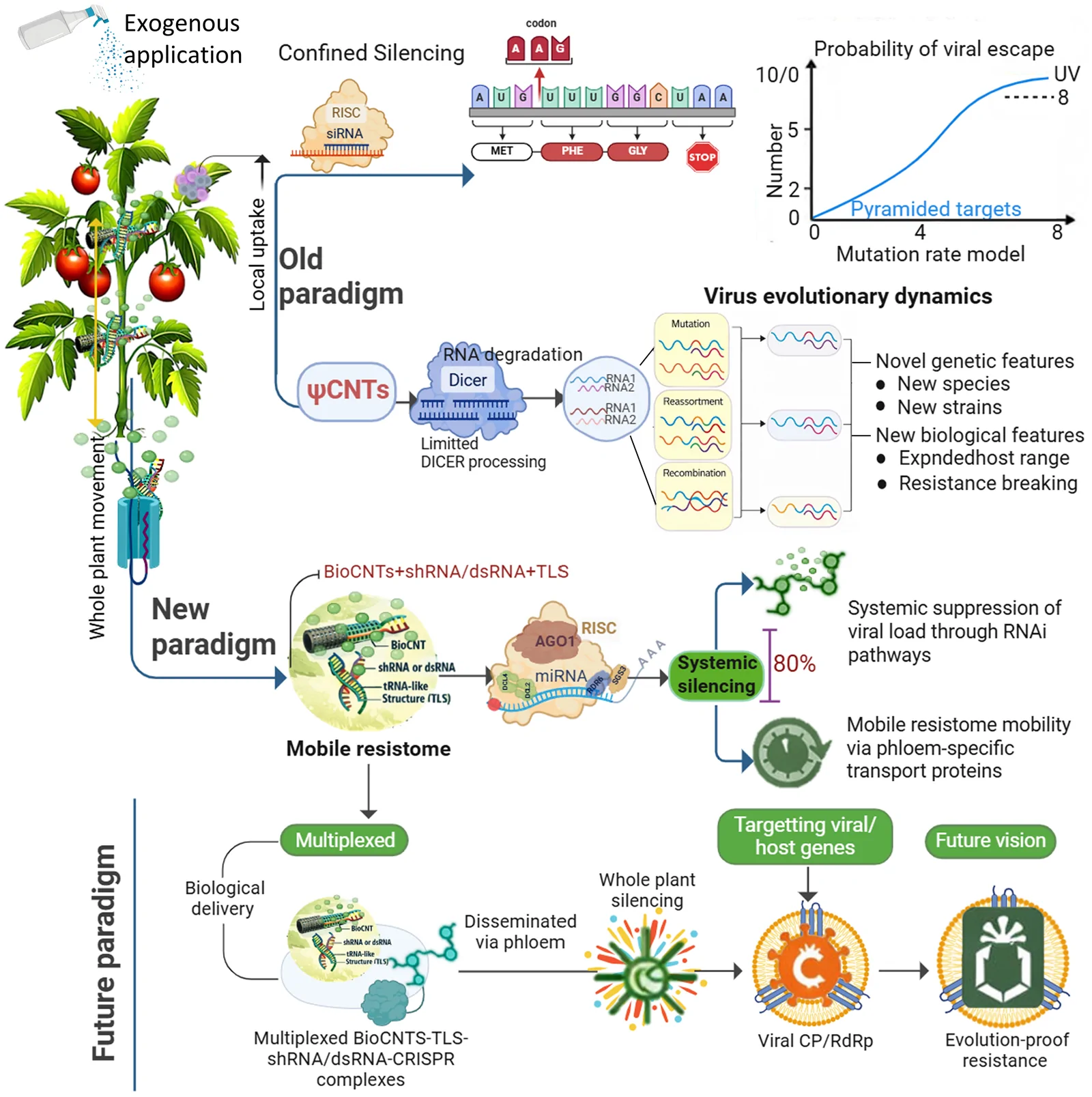
Overcoming the bottlenecks of spray-induced gene silencing (SIGS) via a mobile RNAi platform. Conventional topical application of exogenous double-stranded RNA (dsRNA) is constrained by several physiological and environmental barriers: (i) rapid degradation by extracellular RNases and UV radiation; (ii) inefficient translocation across the plant cuticle and cell wall; and (iii) a lack of systemic mobility. Consequently, the silencing effect remains sequestered at the application site. This localized restriction allows viral populations to persist and replicate in untreated tissues, which serve as reservoirs that facilitate viral evolution and the emergence of resistance. The engineered BioCNTs-TLS system bypasses these limitations through a multi-stage delivery strategy. (i) The BioCNT nanocarrier encapsulates the RNA payload (dsRNA/shRNA), conferring protection against environmental degradation and enhancing endocytosis or direct cellular uptake. (ii) The integration of tRNA-like structures (TLS) facilitates phloem loading, enabling the silencing signal to circumvent cellular boundaries and traverse the plant s vascular architecture. (iii) This transition from a transient, localized intervention to a durable, plant-wide mobile resistome ensures protection in both proximal and distal tissues, effectively minimizing the spatio-temporal windows available for viral escape.

The successful delivery of nucleic acid effectors into the rigid architecture of a plant cell is fundamentally governed by the physicochemical properties of the nanocarrier’s surface (**Figure 3**) (Demirer et al., 2020; Liu et al., 2026). Single-walled carbon nanotubes, characterized by their high aspect ratio and hydrophobic graphitic lattice, require sophisticated non-covalent functionalization to remain stable in aqueous agricultural formulations. The optimization of polymer-wrapping chemistry is reviewed in depth by Sede et al. (2026), who emphasize that the molecular-velcro principle only operates effectively within a narrow window of N/P ratio and ζ-potential; outside this window, either premature cargo release in the apoplast or sequestration and clearance from cytoplasmic compartments is observed (Demirer et al., 2020; Balusamy et al., 2023). The most effective reported strategy involves wrapping the nanotubes in cationic polymers, such as branched polyethyleneimine, which creates an electrostatic molecular velcro that binds the negatively charged phosphate backbone of short hairpin RNA (shRNA). This wrapping process must be meticulously calibrated: the nitrogen-to-phosphate (N/P) ratio determines not only the loading capacity of the carrier but also the zeta potential of the complex, which in turn governs the initial interaction with the negatively charged hemicellulose matrix of the plant cell wall. The N/P range of 10:1 to 20:1 listed in **Table 2** corresponds to the working window reported in the Demirer et al. (2020) experimental system; reader-altering caveats about the relationship between N/P ratio and ζ-potential for plant-relevant delivery have been systematically documented earlier (Sede et al., 2026). A summary of these interfacial engineering parameters and their biological consequences is provided in **Table 2**. Within the working window of the molecular velcro, the shRNA cargo is protected from the rigorous enzymatic environment of the apoplast, where extracellular ribonucleases can degrade naked RNA molecules in less than 120 minutes (Balusamy et al., 2023; Mat Jalaluddin et al., 2023). Critically, the reliance on electrostatic binding presents a significant thermodynamic trade-off between protective sequestration and intracellular availability. If the binding affinity is excessively high, the BioCNT-RNA complex may fail to dissociate within the cytoplasm, effectively locking the genetic payload and preventing its entry into the RNA-induced silencing complex. It is crucial to investigate the use of stimuli-responsive polymers that undergo conformational changes in response to the cytosolic pH gradient to trigger an active unloading mechanism. Whether stimuli-responsive PEI derivatives or alternative lipid-fusion-driven unloading mechanisms will improve in planta cargo release has not yet been experimentally demonstrated; the question is identified by Sede et al. (2026) as one of the field’s most significant unresolved mechanistic bottlenecks.

## 7 The apoplast-symplast barrier: navigating the lipid bilayer

Once the BioCNT-shRNA complex has successfully navigated the cell wall, it encounters the second and most significant physiological barrier-the plasma membrane (Demirer et al., 2020; Liu et al., 2026). Unlike traditional delivery methods like biolistics, which physically rupture the membrane, or Agrobacterium-mediated transformation, which requires biological hijacking, BioCNTs leverage their unique geometry for needle-like penetration or potentially energy-dependent endocytic pathways (Meena et al., 2021; Safdar et al., 2022). The small diameter of SWCNTs (~1 nm) allows them to pierce the lipid bilayer with minimal disruption to the membrane potential, a process facilitated by the longitudinal orientation of the nanotubes during movement (Husen and Siddiqi, 2014; Demirer et al., 2020). Inside the cytoplasm, the BioCNT serves as a protective reservoir, maintaining the structural integrity of the shRNA pyramid until it can be processed by Dicer-like 4 enzymes into the 21-nucleotide small interfering RNAs (siRNAs) required for systemic silencing (Brosnan et al., 2026; Liu et al., 2026). In this context, the assumption that penetration is purely mechanical ignores the potential for lipid-nanotube fusion and the role of membrane-resident proteins in facilitating translocation. A deeper biophysical exploration of the lipid-nano interface is required to explain why certain SWCNT chiralities exhibit significantly higher translocation speeds, potentially suggesting an unrecognized receptor-mediated entry mechanism.

The transition from the apoplastic space (the extracellular matrix) to the symplastic interior (the cytoplasm) is the most critical phase for cargo preservation (Dubrovina and Kiselev, 2019; Zhu et al., 2022). In the apoplast, the BioCNT-RNA complex is exposed to a variety of secreted enzymes and varying pH levels that can compromise the cargo (An et al., 2022; Brosnan et al., 2026). By achieving rapid symplastic entry, the BioCNT platform ensures that the stoichiometry of the delivered RNA is sufficient to initiate a robust antiviral response (Demirer et al., 2020; Liu et al., 2026). Reported viral suppression efficiency in published nanocarrier-SIGS studies ranges widely (40-95%) depending on virus species, host, nanoparticle composition, application method, and assay readout; representative values from primary peer-reviewed sources are summarised in **Table 3**, alongside their measurement readout (symptom severity, viral titre, transcript level) and the specific host-virus pair used in each study (Mat Jalaluddin et al., 2023; Liu et al., 2026). The efficiency of the apoplastic bypass is the primary metric of a successful nanobionic delivery system. Subsequently, the temporal dynamics of this bypass should be focused to determine if a secondary application-the Nanoprimer strategy can temporarily saturate the apoplastic clearance mechanisms to further boost the bioavailability of the primary cargo.

### 7.1 Mechanistic basis of BioCNT internalization in plant cells

The internalization of BioCNTs into intact plant cells is a multi-stage process that bypasses the need for traditional biolistic or *Agrobacterium*-mediated transformation (Jiang et al., 2021; Ahmad, 2023). Once inside, the BioCNT-RNA complex must navigate the intracellular environment and release its cargo in a location accessible to the silencing machinery (Zhu et al., 2022; Haghighi et al., 2024). This level of molecular precision allows for internalization of 70–80% of siRNA-SWNT complexes within 6 hours of administration to the abaxial leaf surface; in *N. benthamiana*, the SWNT carrier extended the apparent residence time of siRNA inside plant cells by approximately 12 hours compared to naked siRNA, with *GFP* silencing observed for 3–7 days post-application (Balusamy et al., 2023). The values are species-, tissue-, and application-method-specific; an extended cargo-residence time of more than 48 hours, as occasionally reported, has not been independently reproduced in peer-reviewed literature outside a narrow set of controlled conditions and should therefore be treated as an upper-bound observation rather than a deployable platform property. The assumption that simple mechanical penetration is the sole driver of CNT uptake is likely an oversimplification of a more complex endocytic or lipid-fusion mechanism. Future studies using super-resolution microscopy are required to map the exact trajectory of BioCNTs from the leaf surface to the phloem companion cells to optimize delivery kinetics.

The mobile resistome must be able to intercept viruses that utilize different systemic movement strategies (Wilson, 1993; Nicaise, 2014). Some viruses, like Tobacco mosaic virus (TMV; *Tobamovirus tabaci*), move primarily through the phloem, while others may utilize the xylem or move cell-to-cell through the epidermis (Wilson, 1993; Tatineni and Hein, 2023). The TLS-shRNA platform is particularly effective against phloem-mobile viruses, as the silencing signal is localized within the same vascular conduit that the virus must use for colonization (Biedenkopf et al., 2020; Liu et al., 2026). For viruses with more diverse movement patterns, the use of BioCNTs to achieve broad epidermal internalization ensures that the mobile resistome remains a formidable barrier regardless of the pathogen’s trajectory (Demirer et al., 2020; Jiang et al., 2021). The phloem-centric nature of the TLS motif may be a double-edged sword, potentially leaving non-vascular tissues like the epidermis or root cortex with lower levels of protection. Future pan-tissue platforms may need to incorporate multiple trafficking motifs such as small peptide conjugates to ensure that the resistome is truly global in its coverage.

Beyond simple electrostatic binding, the introduction of secondary coatings such as single-stranded DNA (ssDNA) or specialized surfactants like sodium dodecyl sulfate can further refine the nanocarrier’s bio-identity (Husen and Siddiqi, 2014; Demirer et al., 2020). These coatings act as surfactants that prevent van der Waals-driven aggregation, ensuring that the SWCNTs maintain their needle-like geometry, which is essential for bypassing the 5–20 nm pore size exclusion limit of the plant cell wall (Mukherjee et al., 2016; Safdar et al., 2022). Experimental data from tomato models indicate that ssDNA-wrapped BioCNTs exhibit a 2.5-fold increase in cellular internalization compared to those wrapped in synthetic polymers, suggesting that the bio-mimetic nature of the DNA coating reduces the plant’s innate recognition of the carbon structure as a xenobiotic (Demirer et al., 2020; Liu et al., 2026). Carbon-nanotube association has been reported to extend the apparent residence time of delivered small RNAs by approximately 12 h relative to naked siRNA in *N. benthamiana* (Balusamy et al., 2023); broader durability in field-relevant crop hosts has not been demonstrated. The transition from synthetic to biomimetic coatings highlights the shift toward soft nanotechnology in agricultural applications. However, the long-term metabolic fate of these DNA-based coatings must be evaluated to ensure they do not interfere with the host’s endogenous nucleotide pools or inadvertently trigger pattern-triggered immunity pathways.

## 8 The protein corona in planta: the secret interface

By analogy with the well-characterised protein-corona phenomenon in mammalian nanomedicine, hypothetical adsorption of apoplastic host proteins onto BioCNTs administered to plants has been proposed as the soft interface that would, in principle, govern downstream cellular recognition and trafficking (Mat Jalaluddin et al., 2023; Sede et al., 2026). However, we emphasise that, in stark contrast to the mammalian case, there is presently no peer-reviewed characterisation of either the compositional identity or the functional impact of any plant-derived BioCNT corona; the corona discussion is therefore presented here as a hypothetical, design-relevant framework rather than as established mechanism. As a consequence, no engineering rules are yet derivable for plant corona composition, and statements that design-defined coronas can be programmed *a priori* should not be read as platform properties. Within this hypothetical framework, hypothetical adsorption of cell-wall-modifying enzymes might facilitate passage through the hemicellulose matrix, and hypothetical binding of defence-related proteins might trigger sequestration and clearance; both effects are presented here as conceptual possibilities that await experimental verification (Mat Jalaluddin et al., 2023; Sede et al., 2026).

If researchers could eventually pre-engineer the BioCNT surface to selectively adsorb facilitator proteins from the apoplast, such a programme would require first characterising which apoplastic proteins bind the carrier under which physiological conditions-a step that has not yet been taken in planta (Sede et al., 2026). The hypothesis that high-density shRNA loading provides a steric shield that minimises non-specific protein adsorption, preserving the accessibility of the trafficking signals, is presently uncorroborated by plant-specific experimental evidence and should therefore be treated as a working hypothesis rather than a design specification. Likewise, the speculation that the protein corona could jam the vascular highway is presented here as a conceptual caveat motivating careful empirical design, rather than as a documented biochemical phenomenon. Achieving a sophisticated understanding of RNA-protein docking under these conditions will require measurements of corona composition in apoplastic fluid and sieve-element protein extracts from BioCNT-treated vs. untreated plants; such measurements do not currently exist for any plant system. This conceptual framework should be read alongside the earlier caveat that the entire protein-corona discussion is hypothetical until experimental plant-corona data are available.

## 9 The spray-to-edit paradigm: transient genome tuning

A burgeoning application of the BioCNT platform is the transient delivery of genome-editing components, such as CRISPR-Cas9 ribonucleoproteins or messenger RNA (mRNA) (Ahmar et al., 2021; Nadakuduti and Enciso-Rodríguez, 2021). Unlike stable transgenic editing, spray-to-edit utilizes the nanocarrier to achieve targeted DNA modifications in somatic tissues that persist only for the duration of the growing season (Touzdjian Pinheiro Kohlrausch Távora et al., 2022; Ahmad, 2023). SWCNTs have demonstrated the unique ability to carry these large, complex payloads into intact cells, where they can transiently modify genes associated with viral susceptibility or abiotic stress tolerance (Liu et al., 2019; Demirer et al., 2020). This allows for the real-time tuning of a crop’s physiology in response to immediate environmental threats, such as a sudden drought or a localized viral outbreak, without the need for permanent, heritable changes (Nadakuduti and Enciso-Rodríguez, 2021; Daniel et al., 2024). Herein, the prospect of seasonal genome tuning marks a qualitatively distinct approach from the one-and-done philosophy of plant breeding. The primary hurdle remains the on-target efficiency; unlike shRNAs which operate through a catalytic silencing mechanism, CRISPR-based editing requires the delivery of a critical mass of RNPs to the nucleus, a feat that currently pushes the limits of carbon nanotube loading density.

Should future studies demonstrate stable, phloem-mobile CRISPR-Cas RNP delivery via BioCNT-TLS, true systemic heritable editing could in principle be realized. At present, however, this remains a conceptual possibility; the limited tissue range of current vascular delivery systems and the lack of demonstrated germline editing in crops by nanoparticle-RNP make this a long-term research objective rather than an imminent capability (Mach, 2016; Zhang et al., 2016). This would allow for the modification of future growth patterns from a single topical application, essentially updating the plant’s genetic software in mid-season (Daniel et al., 2024; Chen et al., 2025). This flexibility is essential for responding to breakout viral strains that render existing resistance obsolete, providing a non-heritable path to enhanced resistance that may be more acceptable to global regulatory bodies (Dietz-Pfeilstetter et al., 2021; Ahmad, 2023). Systemic editing is the logical conclusion of the nanobionic revolution, yet it raises profound safety and ethical questions. Hypothetically, integrating a protease- or self-cleaving peptide-triggered Cas9 degradation motif into the cargo would allow transient editing activity. However, this concept has not been demonstrated in plants and should be evaluated as a future engineering target.

## 10 TLS motifs, vascular loading, and systemic mobility

**Figure 4:**
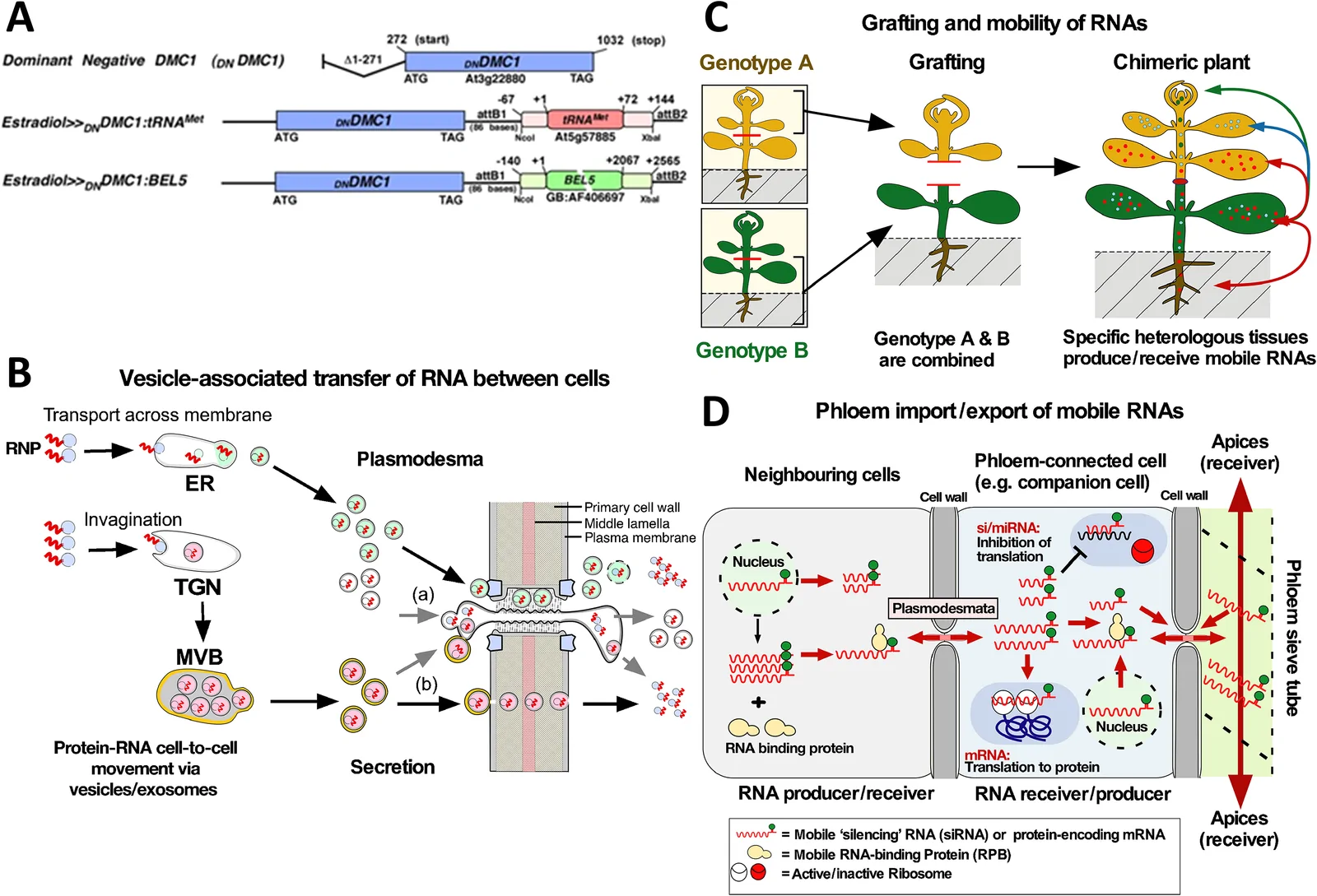
TLS-Mediated phloem loading and systemic transport. **(A)** Secondary structure of the tRNA-like structure motif highlighting loops required for protein binding (Zhang et al., 2016). **(B)** Mechanistic view of the companion cell to sieve element interface, showing BioCNT-TLS transport across plasmodesmata (Kehr and Kragler, 2018). **(C)** Macro-view of source-to-sink movement of the silencing signal from sprayed leaves to distal roots and apical meristems (Kehr and Kragler, 2018). **(D)** Model of RNA intercellular transfer into and from the phloem vessels (sieve tubes) via plasmodesmata and its effect on protein translation in receiving cells (Kehr and Kragler, 2018).

The transition of a localized RNAi signal into a systemic mobile resistome is predicated on the successful entry of the silencing effector into the phloem, the plant’s sophisticated vascular tissue responsible for the long-distance translocation of photoassimilates and signaling molecules (**Figure 4**) (Kehr and Kragler, 2018; Liu et al., 2026). In traditional spray-induced gene silencing, the movement of double-stranded RNA (dsRNA) is often restricted to the apoplast or localized symplastic clusters due to the lack of specific loading motifs (Cagliari et al., 2019; Rank and Koch, 2021). The BioCNT-TLS platform overcomes this by utilizing tRNA-like structures as molecular passports that are recognized by the phloem loading machinery, specifically within the companion cells of the minor veins (Mach, 2016; Zhang et al., 2016). This active recruitment into the sieve elements allows the exogenous RNA to follow the pressure-driven bulk flow from source tissues (sprayed leaves) to sink tissues (roots, young leaves, and floral organs), establishing a plant-wide immune state (Zhang et al., 2009; Liu and Chen, 2018). The specific molecular interactions and physiological stages of this journey are mapped in **Table 3**. The reliance on phloem-mediated transport introduces a physiological dependency where the efficacy of the biopesticide is tied to the plant’s metabolic rate and hydraulic conductivity. In this regard, it is critical to examine how environmental stressors such as drought or extreme temperatures which reduce phloem flow, might inadvertently create immune deserts in distal tissues where the viral interception is most needed.

The phloem microenvironment is characterized by a relatively high pH (~8.0–8.5) and a high concentration of specialized RNA-binding proteins that stabilize mobile RNAs during their journey (Kehr and Kragler, 2018; Liu and Chen, 2018). Functionalized BioCNTs provide an additional layer of protection in this environment, shielding the RNA from alkaline hydrolysis and the diverse array of ribonucleases present in the phloem sap (Demirer et al., 2020; Jiang et al., 2021). Unlike viral genomes that must encode their own movement proteins to dilate plasmodesmata and facilitate vascular entry, the BioCNT-TLS complex utilizes endogenous trafficking pathways, potentially reducing the energetic cost of systemic mobilization for the host plant (Zhang et al., 2016; Liu et al., 2026). This neutral pathway description ensures that the antiviral signal is distributed with the same efficiency as the plant’s own developmental signals (Zhang et al., 2009; Mach, 2016). While the use of endogenous pathways minimizes host stress, the potential for competition between synthetic TLS-RNAs and natural signaling mRNAs for a finite number of RNA-binding protein (RBP) chaperones remains a significant unknown. Future research must determine the carrying capacity of the phloem for exogenous genetic payloads to avoid saturating the plant’s internal communication network.

The engineering of tRNA-like structures at the 3’ end of shRNA constructs is the definitive innovation that enables long-distance transport within the BioCNT platform (Liu et al., 2026). TLS motifs are conserved RNA secondary structures that mimic the L-shape of functional tRNAs and are capable of being aminoacylated by host enzymes (Zhang et al., 2009; Zhang et al., 2016). In the context of the mobile resistome, these structures serve as high-affinity recognition sites for phloem-specific proteins, such as Phloem Protein 2 and other ribonucleoprotein chaperones (Mach, 2016; Kehr and Kragler, 2018). These interactions facilitate the movement of the BioCNT-RNA complex through the plasmodesmata connecting companion cells to sieve elements, effectively crossing the symplastic/vascular boundary (Zhang et al., 2016; Liu and Chen, 2018).

Beyond simple recognition, the TLS motif may also influence the unloading kinetics of the RNA in sink tissues (Zhang et al., 2016; Kehr and Kragler, 2018). Upon reaching the destination, the aminoacylation state of the TLS or its interaction with specific sink-localized RBPs may trigger the release of the shRNA from the sieve elements into the surrounding parenchyma (Zhang et al., 2009; Liu and Chen, 2018). This targeted unloading ensures that the silencing signal is concentrated in the most vulnerable regions of the plant, such as the shoot apical meristem, where viruses often aggregate for systemic colonization (Nicaise, 2014; Liu et al., 2026). The use of BioCNTs as carriers facilitates this process by maintaining the structural integrity of the TLS-shRNA complex until it reaches these distal unloading sites (Demirer et al., 2020; Safdar et al., 2022). The unloading mechanism is arguably more complex than phloem loading, as it must distinguish between transient transport and permanent tissue deposition. Determining whether a sink-specific TLS variant could further optimize the delivery of the resistome to high-value organs such as developing fruits remains a frontier for precision agriculture.

## 11 Current limitations and unknowns

TLS-mediated long-distance mRNA transport has been characterised predominantly in *Arabidopsis thaliana* and heterograft systems BioCNT-shRNA systemic mobility, by contrast, has been demonstrated in tomato and *N. benthamiana* (Liu et al., 2026). Whether TLS motifs retain their trafficking function in monocots, woody perennials, or cold-tolerant field crops remains untested. Furthermore, reported SIGS performance under controlled conditions does not necessarily translate to the field. UV exposure, rainfall wash-off, cuticle thickness, leaf age, canopy architecture and plant physiological status have each been documented to attenuate topically applied dsRNA efficacy. Dose-response relationships for BioCNT-TLS formulations under these variables have not yet been published. In addition, persistent, phloem-limited pathogens (e.g., *Candidatus* Liberibacter spp., Citrus tristeza virus (CTV; *Closterovirus tristezae*)) encode suppressors of host RNA silencing that may attenuate SIGS-derived responses. The interaction between BioCNT-shRNA constructs and viral VSRs has not been characterised; this should be considered when targeting such pathogens.

Liu et al. (2026) report that distal shRNA accumulates above the silencing threshold at ~14 days post-application in *N. benthamiana*, with 50-60% reduction in target transcripts in systemic leaves. Against fast-moving epidemics especially poleroviruses such as Cotton leafroll dwarf virus (CLDV; *Polerovirus CLDV*), this delay represents a vulnerability window during which the plant is unprotected. Pre-emptive prophylactic deployment, or staggered re-application, may be required; integrated scheduling with respect to vector pressure has not been modelled.

## 12 RNA stability in vascular transport

The half-life and structural integrity of sprayed or carrier-formatted RNA determines whether enough intact effector reaches apoplastic, symplastic, and phloem targets to produce a measurable silencing response. Naked long dsRNA applied to foliar surfaces is rapidly degraded, with detectable molecule loss observable within hours of application; in Arabidopsis, 22-bp siRNA infiltrated into leaf tissue could not be recovered beyond 6 hours post-application (hpa) when applied without stabilization (Bennett et al., 2020). Environmental UV exposure further accelerates this decay, with visible agarose-gel degradation detectable after as little as 30 minutes of UV irradiation (He et al., 2022). In soil, microbial nucleases drive exponential degradation: dsRNA can be fully degraded in agricultural soils within 2–3 days of application, with neither dose, length, nor molecular structure providing measurable protection against biotic decay (Bachman et al., 2020; Romeis and Widmer, 2020). These observations motivated the development of carrier systems- cationic polymers, layered double hydroxide (LDH/BioClay), guanylated polymers, chitosan nanoparticles, and carbon nanotube scaffolds-whose principal role is to physically shield the RNA backbone from direct contact with solvent-exposed RNases and from photo-oxidative damage.

Once RNA enters the plant interior, it faces a second, distinct set of stability challenges at the apoplast-symplast interface and within the vascular stream. Phloem sap contains numerous nucleases and is broadly nearer neutral to slightly alkaline (pH ~7.0–8.0), whereas apoplastic fluid is more acidic and contains secreted RNases that can shorten functional half-life considerably (Kehr and Kragler, 2018). Systemic mobility across long distances therefore depends on whether the RNA is recruited to a phloem-mobile ribonucleoprotein complex. tRNA-like structures, originally identified at the 3′-termini of certain plant positive-strand RNA viruses and subsequently shown to be present in endogenous plant mRNAs, are sufficient to mediate phloem recruitment and graft-junction transport of mRNA in *Arabidopsis thaliana* (Zhang et al., 2016; Wang et al., 2021). The mechanism involves binding of TLS-bearing transcripts to phloem small-RNA binding proteins such as *CmPSRP1* in *Cucurbita maxima*, which selectively recognizes 25-nt single-stranded RNAs and mediates cell-to-cell trafficking through plasmodesmata (Yoo et al., 2004; Zhang et al., 2009). Beyond TLSs, secondary stem–bulge–stem–loop structures in pre-miRNA precursors have been demonstrated to provide a parallel route into sieve-element unloading, signifying that multiple distinct motifs can recruit overlapping RNA-binding protein partners (Wang et al., 2021).

For BioCNT-TLS complexes, the principal stability contribution is therefore two-fold: (i) protection of RNA cargo against apoplastic and surface nucleases prior to internalization, and (ii) presentation of TLS motifs (engineered into the cargo) that promote phloem loading and sustained movement into sink tissues. A critical unresolved question is the half-life of carrier-formatted dsRNA/shRNA inside the phloem stream-quantification of this parameter with plant species, cargo length, and carrier chemistry is currently a research gap. Similarly, the regulatory consequences of RNA compartmentalization remain poorly characterized: TLS-mediated entry may route the cargo into translational recycling rather than RISC loading, producing divergent silencing outcomes across species. A tiered approach combining (a) physical protection assays, (b) cellular uptake assays (confocal microspectroscopy), and (c) phloem exudate sampling (e.g., aphid stylectomy, EDTA-facilitated exudation) is therefore needed to evaluate any candidate BioCNT-TLS formulation rigorously.

## 13 Signal amplification and the secondary siRNA wave

A unique feature of the plant RNAi machinery is the ability to amplify the silencing signal through the action of RNA-dependent RNA polymerases, particularly RNA-dependent RNA polymerase 6 (RDR6) (Mezzetti et al., 2020). When the BioCNT-TLS platform delivers a primary shRNA, it is processed by Dicer-like 4 enzymes into 21-nucleotide siRNAs (Brosnan et al., 2026; Liu et al., 2026). These primary siRNAs then guide the RISC complex to target viral mRNA, but they also serve as primers for RDR6 to synthesize new dsRNA from the cleaved viral templates (Dubrovina and Kiselev, 2019; Biedenkopf et al., 2020). This secondary dsRNA is then processed into a diverse wave of secondary siRNAs, which spreads ahead of the primary infection front, creating a robust and persistent state of immunity (Wang and Jin, 2017; Ray et al., 2022). This RDR-mediated amplification transforms a localized application into a living molecular defense that scales with the threat. However, if the initial BioCNT-mediated delivery is too low to reach the threshold of initiation, the secondary wave may never materialize, highlighting the critical importance of nanocarrier penetration efficiency.

**Figure 5:**
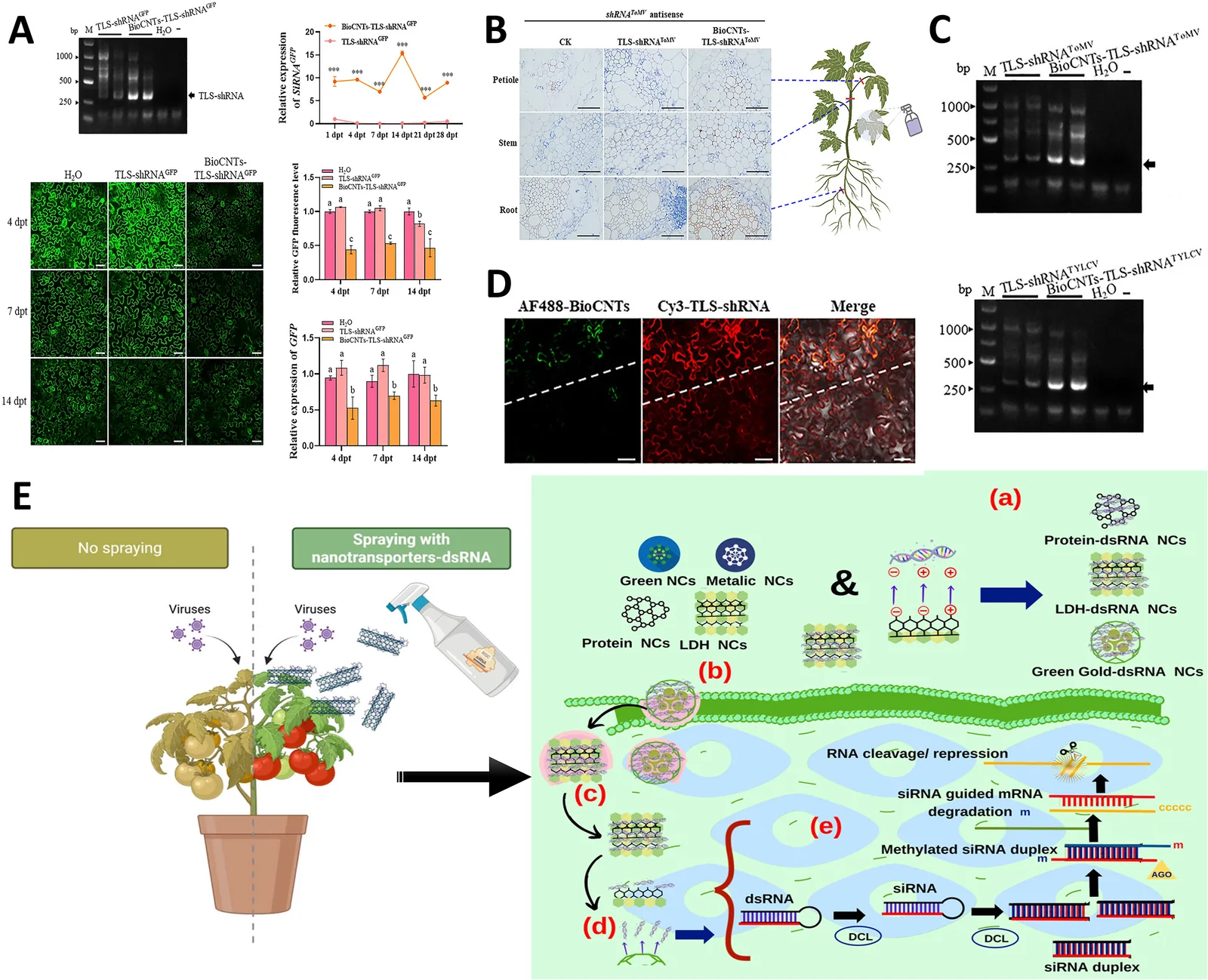
Systemic silencing mechanism and sirna dynamics. **(A)** Nanocarrier-mediated delivery and endocytic entry. Gel electrophoresis of TLS-shRNA*^GFP^* in treated tomato leaves (Liu et al., 2026). Stem-loop RT-qPCR of *siRNA^GFP^*accumulation over time (1–28 dpt), and lower panel shows confocal imaging of BioCNTs and TLS-shRNA in planta (Liu et al., 2026). **(B)** *BioCNTs* enhance TLS-shRNA stability/mobility for virus prevention. *In situ* hybridization of *shRNA^ToMV^*in tomato roots/stems/petioles (scale bar = 15 µm) (Liu et al., 2026). **(C)** siRNA levels (ToMV/TYLCV) over 1–28 days post-treatment via gel electrophoresis and RT-qPCR (Liu et al., 2026). **(D)** Confocal imaging of BioCNTs and TLS-shRNA in cells (scale bar = 50 µm) (Liu et al., 2026). **(E)** Nanocarrier-mediated viral disease management. **(a)** dsRNA binds nanocarriers (covalent/non-covalent) for delivery. **(b)** Nanocarriers enter cells via endocytosis (membrane interaction/lysis). **(c)** Endosome escape releases dsRNA to cytoplasm. **(d)** dsRNA release activates gene silencing (siRNA production). **(e)** DCL cleaves dsRNA → siRNA duplex (methylated by HEN); AGO binds siRNA to silence viral RNA (Vasquez-Gutierrez et al., 2025). *(TLS, tRNA-like structure; BioCNTs, biocompatible carbon nanotubes; DCL, Dicer-like; siRNA, small interfering RNA; dsRNA, double-stranded RNA; HEN, HUA ENHANCER 1; AGO, Argonaute; NCs, nanocarriers)*.

The spatial distribution of these secondary siRNAs is dictated by both the movement of the BioCNT-TLS complex and the subsequent cell-to-cell spread of the 21-nt and 24-nt siRNAs through plasmodesmata (Kehr and Kragler, 2018; Liu and Chen, 2018). Studies using the BioCNT platform have shown that this secondary wave can maintain silencing in distal tissues for several weeks, even as the original nanocarriers are degraded or diluted by plant growth (**Figure 5**) (Liu et al., 2026). This suggests that the BioCNT-TLS system acts as a starter culture for the plant’s own immune system, initiating a self-sustaining genomic defense that does not require constant reapplication of the biopesticide (Rank and Koch, 2021; Xi et al., 2025).

## 14 Spatial mapping of the global mobile resistome

The efficacy of the systemic resistome is measured by its ability to suppress viral titers across the entire plant body. In tomato models treated with BioCNT-TLS-shRNA, peak distal silencing is typically achieved 7 to 14 days post-application, with significant knockdown observed in the youngest developing leaves (Liu et al., 2026). This spatial distribution pattern is highly correlated with the source-to-sink orientation of the vascular system, where the highest concentrations of siRNAs are found in tissues with the highest metabolic demand (Zhang et al., 2009; Kehr and Kragler, 2018). By mapping these siRNA gradients, researchers can optimize spray patterns to ensure that the mobile resistome covers the most critical parts of the crop canopy (Ghosh et al., 2023; Chen et al., 2025). Importantly, the current mapping of siRNA gradients is largely two-dimensional, ignoring the complex three-dimensional architecture of mature crop plants.

## 15 Cross-kingdom RNAi: extending the mobile resistome to pathogens

The utility of the systemic mobile resistome extends beyond the boundaries of the plant host to include the management of fungal pathogens and insect vectors through Cross-Kingdom RNAi (Cagliari et al., 2019; Brosnan et al., 2026). In this paradigm, the systemic shRNAs and siRNAs produced within the plant are transferred directly into the feeding pathogen or pest, where they initiate the silencing of essential genes (He et al., 2022; Chen et al., 2025). For fungal pathogens like *Fusarium oxysporum*, the BioCNT platform facilitates the transfer of RNA effectors across the host-pathogen interface, potentially via extracellular vesicles or direct uptake by fungal hyphae (Ray et al., 2022; Xi et al., 2025). This allows for the silencing of genes critical for fungal cell-wall integrity or ergosterol synthesis, providing a sequence-specific alternative to broad-spectrum chemical fungicides (Ray et al., 2022; Touzdjian Pinheiro Kohlrausch Távora et al., 2022). Cross-Kingdom RNAi represents a form of neutral cross-kingdom, where the plant’s own vascular system is transformed into a delivery network for biopesticides. The durability of this approach depends on the inter-species compatibility of the silencing machinery; it must be ensured that the RNA effectors are structurally optimized to resist the diverse range of suppressors of RNA silencing produced by fungi and viruses.

For insect pests, particularly sap-sucking vectors like the whitefly *Bemisia tabaci*, the systemic shRNAs in the phloem sap are ingested during feeding (He et al., 2022; Liu et al., 2026). Once inside the insect’s midgut, these RNAs must survive a challenging environment characterized by high pH (often 9.0–10.0 in lepidopterans) and the presence of potent gut nucleases (He et al., 2022; Rodriguez Coy et al., 2022). The BioCNT-TLS platform addresses this by ensuring a high concentration of systemic RNA throughout the vascular architecture, increasing the probability that a lethal dose reaches the insect’s internal tissues (Biedenkopf et al., 2020; Liu et al., 2026). By targeting genes essential for viral transmission or insect survival, this dual-action approach silencing the virus in the host while simultaneously disrupting its vector offers a comprehensive solution to the viral arms race (He et al., 2022; Vasquez-Gutierrez et al., 2025). Targeting the vector-pathogen interface is the most significant strategic advantage of the mobile resistome. However, the environmental fate of these trans-kingdom signals must be rigorously assessed; we must use high-throughput bioinformatic screening to ensure that these shRNA pyramids do not inadvertently target beneficial pollinators or soil microbiota that share the same genomic motifs.

## 16 Delivery routes and scalability

Three principal delivery routes have been evaluated for topically applied RNAi in crop protection, with a fourth route (trunk injection) reserved largely for woody perennials (**Table 4**). Foliar spray is currently the most scalable route because (i) it leverages existing spray equipment, (ii) it requires the smallest reformulation of agronomic practice, and (iii) regulatory frameworks for foliar biopesticides (including EPA FIFRA Section 3 registrations for dsRNA products such as Ledprona) already exist (He et al., 2022). The principal limitation is delivery efficiency: only a small fraction (often <1% in classical chemical spraying) of applied active substance reaches the intended cuticular, stomatal, or phloem target (Singewar and Fladung, 2023). LDH-clay carriers significantly improve leaf-surface adhesion, reduce wash-off by rain and dew, and extend the protection window by 2–4× compared with naked dsRNA (Fletcher et al., 2020; Niño-Sánchez et al., 2022). For piercing-sucking pests, the additional transport through the plant vascular system is required; MgAl-LDH and MgFe-LDH composites have recently been shown to enhance dsRNA translocation from stem to distal leaves in pomelo, increasing nymph mortality in *Panonychus citri*, *Diaphorina citri*, and *Aphis gossypii*-providing proof of concept that carrier-mediated SIGS can reach stylet-feeding insects through vascular spreading (Cheng et al., 2024).

**Table 4:** Plant SIGS/RNAi delivery routes: action targets, scalability, and notable results.

Route	Action target	Scalability	Notable results	Citation
Foliar spray	Chewing/surface-feeding insects; foliar pathogens; some phloem-feeders via SIGS distribution	High (compatible with existing spray equipment)	dsRNA against *Leptinotarsa decemlineata* actin provides protection on potato foliage; BioClay extended antiviral protection in Nicotiana tabacum infected with Cucumber mosaic virus (CMV; Cucumovirus CMV) from ~5 days (naked dsRNA) to ~20 days	(Worrall et al., 2019; Fletcher et al., 2020; Niño-Sánchez et al., 2022)
Root irrigation/drench	Soil-borne pathogens; vascular pathogens; hemipterans feeding on xylem	Medium-high (under greenhouse/hydroponics); lower in mature field soil due to microbial dsRNase activity	dsRNA can be taken up by roots and, when formatted within BioCNT-TLS, may distribute systemically via xylem; chitosan-dsRNA complexes have shown protection against root pathogens	(Scarpin et al., 2023)
Seed treatment/priming	Early-season insect pests; seedling pathogens	High (lowest total dose; systemic from germination)	Limited studies, but reported to produce seedling-stage protection in cereals against aphids and wireworms	(Dietz-Pfeilstetter et al., 2021; He et al., 2022)
Trunk injection	Xylem-feeding insects; phytoplasmas; some vascular pathogens	Low for row crops; medium for orchards and forestry	Dalakouras et al. demonstrated dsRNA delivery to the xylem of woody hosts and systemic spread; particularly relevant to perennial crops like grapevine, citrus, and apple	(Dietz-Pfeilstetter et al., 2021)

At the spray-application layer, recent work has begun to test whether nanocarrier-facilitated delivery of multi-gene-targeting dsRNA formulations can extend this rationale to phloem-feeding Hemipterans of major economic importance. Alghadab and Velasco (2026) report nanoparticle-enhanced, multi-target dsRNA sprays against *Myzus persicae*, providing one of the few current experimental demonstrations that nanocarrier chemistry can simultaneously deliver dsRNAs to multiple essential transcripts in a single aphid species. The result establishes the feasibility of carrier-mediated multi-target SIGS at the aphid-plant interface, but is, at present, a proof-of-concept demonstrated only in *M. persicae*; whether the same nanocarrier-dsRNA pairing retains potency in other aphid species, in additional Hemipteran vectors, or under open-field spray regimes remains to be established.

Several practical considerations remain underdeveloped. First, dose-response relationships under field conditions are not yet well characterized; most published reports use laboratory or greenhouse concentrations orders of magnitude above what commercial SIGS products are likely to deploy. Second, retention time on the leaf surface under realistic UV, rainfall, and dew conditions requires more rigorous quantification across multiple climate zones. Third, the volume/mass ratio of carrier to dsRNA in any deployed formulation must be tuned to (i) keep the cost per hectare competitive with conventional agrochemicals, (ii) avoid excessive metallic or polymeric load in the soil, and (iii) preserve colloidal stability of the spray suspension (Fletcher et al., 2020; Romeis and Widmer, 2020; Rank and Koch, 2021). Until these parameters are standardized, foliar spray will remain a research-grade delivery route rather than a deployable agronomic practice-a point widely shared in the field (Bennett et al., 2020; Pugsley et al., 2021; Rank and Koch, 2021).

## 17 Soil ecotoxicology of BioCNTs

Multi-walled carbon nanotubes, single-walled CNTs, and their functionalized derivatives are predicted to enter agricultural soils primarily via (i) direct soil amendment during nano-fertilization or nano-pesticide deployment, (ii) wash-off from foliar application, (iii) litter and root-turnover return, and (iv) co-deposition with biosolids from treated wastewater (Liné et al., 2017). Modeled predicted environmental concentrations for CNTs in European agricultural soils are approximately 35 ng kg⁻¹ in natural/urban soils and up to 12 µg kg⁻¹ in sludge-treated soils, while literature-derived experimental concentrations used in ecotoxicological tests are typically thousands of times higher (10–10,000 mg kg⁻¹) (Adeel et al., 2021b). Closing this gap between predicted and tested concentrations is the most pressing challenge for producing environmentally relevant risk profiles. This three- to five-order-of-magnitude discrepancy is consistent with the broader methodological pattern in terrestrial nano-object risk profiling summarised by Liné, Larue and Flahaut (Liné et al., 2017) and by Adeel et al. (2021b), both of whom emphasise that testing at predicted environmental concentrations, rather than at the mg-kg⁻¹ range, remains the exception rather than the norm for engineered nanocarbons.

MWCNT effects on soil bacterial communities are context-dependent but generally modest. At realistic-to-modest concentrations (10–1000 mg kg⁻¹), most 16S-rRNA-based studies report no significant shifts in overall diversity, although community composition may change (Kerfahi et al., 2015; Mukherjee et al., 2016). At 5000 mg kg⁻¹ of functionalized MWCNTs (fMWCNTs), community composition is significantly altered as visualized by NMDS ordination, with relative-abundance shifts in dominant phyla and a concomitant decrease in soil pH attributable to surface carboxyl/acid groups; this effect was already detectable at 2 weeks and persisted at 8 weeks (Kerfahi et al., 2015). Long-term exposure studies over 1 year show that MWCNTs at 1 mg g⁻¹ dry soil moderately reduce microbial biomass DNA and shift bacterial community profiles, but the magnitude of effects is comparable to those of biochar or industrial carbon black rather than orders of magnitude greater (Ge et al., 2016). Across studies, the strongest determinants of microbial response are CNT surface functionalization, dispersant chemistry, and dose. Importantly, certain soil bacterial phyla demonstrate resilience, recovering to baseline within ~56 days of exposure (Wu et al., 2019).

Earthworms are model detritivores in soil ecotoxicology. CNTs are not highly bioaccumulated by earthworms because of an effective depuration system; in some studies, polycyclic aromatic hydrocarbons attached to CNTs become less bioavailable to worms, suggesting CNTs can sequester co-contaminants rather than increase their uptake (Maurer-Jones et al., 2013; Liné et al., 2017). In contrast, when CNT concentrations are sufficiently high, reproductive endpoints are affected: *Eisenia veneta* exposed via food to 495 mg kg⁻¹ double-walled CNTs produced ~60% fewer cocoons over a 28-day exposure window (Adeel et al., 2021b). Available CNT-earthworm literature remains limited (fewer than ten published studies globally), so reproductive-endpoint no-observed-effect concentrations are not yet well established (Adeel et al., 2021b).

Subsurface nematode reproduction and AMF colonization rates have been reported to decline at high CNT doses, but mechanistic interpretations are complicated by CNT-mediated alterations in soil water-holding capacity and metal availability (Liné et al., 2017). CNTs can act as contaminant vectors: when applied to soil spiked with metals or aged organochlorines (chlordane, DDx), they either reduce co-contaminant accumulation (e.g., MWCNTs decreased chlordane and DDx uptake by 21-80% across zucchini, corn, tomato, soybean) or, in some cases, increase accumulation of certain metals, depending on species and CNT functionalization (De La Torre-Roche et al., 2013; Leroy et al., 2022). This effect must be considered explicitly when designing BioCNT-TLS field trials.

## 18 Biodegradability and end-of-life pathways of BioCNTs

Carbon nanotubes are not biodegradable in the classical microbial sense: their sp²-bonded graphitic backbone is chemically recalcitrant to most enzymatic attack. However, partial oxidative and enzymatic degradation pathways have been characterized, and the resulting half-lives define the long-term environmental persistence of BioCNT-TLS residues.

Horseradish peroxidase, a classical plant-secreted peroxidase, catalyzes biodegradation of SWCNTs in the presence of low H_2_O_2_ (~40 µM) at 4 °C over 12 weeks, with progressive loss of structural integrity detectable by TEM, DLS, gel electrophoresis, mass spectrometry, and UV–vis–NIR spectroscopy (Allen et al., 2008). This HRP mechanism is generalizable: many plant peroxidases and animal peroxidases (myeloperoxidase, eosinophil peroxidase) can degrade carbonaceous nanomaterials, with the reaction proceeding via oxidative shortening from defective sites and unzipping of sidewalls (Kotchey et al., 2013). Among ligninolytic enzymes, manganese peroxidase-but not lignin peroxidase- degrades pristine SWNTs via Mn²^+^ oxidation; notably, oxidized (carboxylated) SWNTs are recalcitrant to MnP because surface carboxyl groups chelate Mn²^+^ at the enzyme’s active site, blocking the catalytic cycle (Zhang et al., 2014). White-rot fungi such as *Phanerochaete chrysosporium* can partially transform pristine and oxidized MWCNTs through excreted laccase and MnP, producing oxidized carbon (C=O, O–H) and shortened tubes, but complete mineralization was not achieved over short timescales (Ma et al., 2019).

Using ¹^4^C-labelled MWCNTs and ¹³C-depleted SWCNTs, Flores-Cervantes et al. estimated an environmental half-life of approximately 80 years under HRP-driven oxidative conditions at ambient H_2_O_2_ exposure-far slower than the “days-to-weeks” originally claimed from qualitative electron-microscopy-based studies (Flores-Cervantes et al., 2014). This discrepancy highlights the methodological importance of using isotopic labelling rather than morphological inspection alone to claim biodegradation.

Regarding implications for BioCNT-TLS deployment, two strategies can mitigate persistence: (i) co-formulating with sacrificial, biodegradable coatings (chitosan, alginate, polylactide, polyhydroxyalkanoates) that themselves degrade in months and release fragmented, defect-rich CNTs that are more susceptible to subsequent enzymatic attack; and (ii) selecting surface chemistries that maximize accessible defect sites (carboxyl- or hydroxyl-rich, but not in combinations that block Mn²^+^ binding) (Kotchey et al., 2013; Zhang et al., 2014). Despite these strategies, the fraction of BioCNT cargo that reaches the soil is likely to persist on timescales of years to decades, underscoring the need for systematic life-cycle assessment of the full delivery system-not just the RNA payload-before any commercial deployment (Flores-Cervantes et al., 2014; Liné et al., 2017).

### 18.1 Long-term soil accumulation and root-uptake knowledge gaps

Half-life data are central to any ecological persistence assessment. Flores-Cervantes et al. (2014) used ¹^4^C-labelled MWCNTs and ¹³C-depleted SWCNTs and estimated an environmental half-life on the order of 80 years under horseradish-peroxidase-driven oxidative conditions at ambient H_2_O_2_ exposure-an order of magnitude slower than the days-to-weeks timescales originally inferred from qualitative electron-microscopy-based studies lacking isotopic mass-balance verification. Surface chemistry controls susceptibility (Zhang et al., 2014) showed that manganese peroxidase partially degrades pristine SWCNTs via Mn²^+^ oxidation, but that carboxylated SWCNTs are recalcitrant because surface carboxyl groups chelate Mn²^+^ at the enzyme active site. Ma et al. (2019) reported that the white-rot fungus *Phanerochaete chrysosporium* can partially transform pristine and oxidised MWCNTs through excreted laccase and MnP, producing oxidised carbon (C=O, O–H) and shortened tubes, although complete mineralisation was not demonstrated over short timescales.

Two engineering strategies can mitigate persistence: (i) co-formulating with sacrificial biodegradable coatings (chitosan, alginate, polylactide, polyhydroxyalkanoates) that themselves degrade within months and release fragmented, defect-rich CNTs more susceptible to subsequent enzymatic attack (An et al., 2022); and (ii) selecting surface chemistries that maximise accessible defect sites, while avoiding combinations that block Mn²^+^ binding (Zhang et al., 2014). Quantitative data on root uptake and re-translocation of residual CNTs originating from a BioCNT-TLS formulation are not yet available. The closest experimental anchors - McGehee et al. (2017), who documented LC-MS-detectable metabolome shifts in tomato fruit following hydroponic MWCNT uptake, and Chen et al. (2015), who used TEM and confocal Raman spectroscopy to localise CNTs inside mature *Brassica juncea* root cells-have not been extended to harvest-mature, fruit-bearing crops sprayed with BioCNT-TLS formulations, nor to multi-season accumulation kinetics. Trophic-transfer bioaccumulation and the corresponding human dietary exposure implications therefore remain unresolved.

Furthermore, the potential for non-target effects of the systemic RNAi signal must be rigorously evaluated (Dietz-Pfeilstetter et al., 2021; He et al., 2022). While the shRNA sequences are designed to be highly specific to viral replicases, the possibility of unintended silencing in beneficial insects or soil microbiota cannot be entirely dismissed (Kah et al., 2021; He et al., 2022). Cross-kingdom RNAi pathways mean that a systemic mobile resistome could theoretically interact with any organism that consumes the plant tissue or shares its vascular environment (Cagliari et al., 2019; Brosnan et al., 2026). Future risk assessments should utilize high-throughput bioinformatic screening and environmental omics to ensure that the delivered RNA pyramids do not disrupt the delicate balance of the agricultural holobiont (Dietz-Pfeilstetter et al., 2021; Chen et al., 2025). Noticeably, the specificity of RNAi is its greatest strength, yet off-target effects in the broader ecosystem remain an aspect of SIGS that is incompletely characterized. Integrating sequence-masking technologies-where the RNA is only activated in the presence of viral proteases could provide an additional layer of ecological safety.

## 19 Non-target organisms risk assessment

Honeybees (*Apis mellifera*) and other pollinators are exposed to nanomaterials during foraging through contact with contaminated pollen, nectar, leaf surfaces, and guttation droplets, as well as through dust deposition on hive materials (Hooven et al., 2019). Direct experimental data on CNTs in honeybees and bumblebees remain scarce; most published pollinator nanomaterial studies have examined titanium dioxide (TiO_2_) or carbon black. Chronic exposure of *Apis mellifera carnica* to nanosized CB and TiO_2_ produced no measurable effects on feeding behavior, survival, or stress-enzyme activities in the brain, suggesting limited toxicity of these particular carbon-based materials at tested doses (Jemec et al., 2016). However, these results cannot be straightforwardly extrapolated to BioCNT-TLS, which is a structurally distinct, sharply anisotropic material with potentially different gut-barrier interactions. To date, BioCNT-specific oral, chronic, and contact toxicity data for *Apis mellifera*, *Bombus* spp., or *Osmia* spp. do not appear to exist in the peer-reviewed literature; the closest available proxies for IPM risk assessment are the 28-day dietary silica-nanoparticle exposure in *Bombus terrestris* (Mommaerts et al., 2012) and the chronic carbon-black/TiO_2_ exposure in *Apis mellifera carnica* (Jemec et al., 2016), both of which establish that at least some inorganic nanoparticles disrupt midgut epithelium or introduce route-specific hazards, while leaving the BioCNT-TLS risk profile unresolved.

Bumblebee (*Bombus terrestris*) dietary exposure to silica nanoparticles (used here as a proxy for inorganic nanocarriers in general) showed that high concentrations (≥34 mg L⁻¹) caused severe midgut epithelial injury and total reproductive failure, although lower concentrations were benign (Mommaerts et al., 2012). Such concentration-dependent effects emphasize the importance of tiered pollinator testing regimens, including acute oral, chronic oral, and contact routes, before any BioCNT-TLS deployment. The widely held assumption that *Apis mellifera* is a sufficient surrogate for wild bee species is not well supported: comparative studies show species-specific differences in oral toxicity for the same chemical across *Bombus terrestris audax*, *Osmia bicornis*, and *Apis mellifera*, with no consistent taxonomic pattern (Heard et al., 2017). BioCNT-TLS-specific data on non-Apis pollinators remain a near-total data gap, an absence made more problematic because the closest published quantum of evidence (Mommaerts et al., 2012), documenting severe midgut epithelial injury and total reproductive failure in *Bombus terrestris* at silica-nanoparticle dietary concentrations of ≥34 mg L⁻¹ -already demonstrates that nanoparticle effects on wild-bee reproduction can differ quantitatively and qualitatively from those observed in honeybees.

Predatory mites, lady beetles (*Coleomegilla maculata*), parasitoid wasps, and lacewings constitute the natural-enemy community that supports IPM programs. *Coleomegilla maculata* has been used as a representative beneficial beetle to assess the off-target RNAi risk of foliar dsRNA targeting *Diabrotica virgifera*; bioinformatic off-target screening with tools such as OfftargetFinder has been used to identify siRNA sequences that minimize homology against lady-beetle transcripts. For BioCNT-TLS, parallel risk analyses using dsCheck (Naito et al., 2005). Si-Fi (Lück et al., 2019) and E-RNAi (Horn and Boutros, 2010) should be applied to all conserved target regions before any field deployment.

Sprayed dsRNA can be taken up by chewing and sap-feeding insects through the cuticle and gut, but the mechanism of uptake is taxon-specific. In Coleoptera (e.g., *Leptinotarsa decemlineata*, *Diabrotica virgifera*), SID-1-like transmembrane channel orthologues mediate dsRNA uptake and are required for robust RNAi (Nitnavare et al., 2021; He et al., 2022). Diptera (including D*rosophila melanogaster* and Bactrocera spp.) lack SID-1 orthologues and rely instead on clathrin-dependent endocytosis for dsRNA uptake (Saleh et al., 2006; Swevers et al., 2013). Lepidoptera and Orthoptera vary interspecifically in dsRNA sensitivity, with some species requiring high doses to achieve measurable knockdown-a disadvantage that may limit the operational target range of BioCNT-TLS (Swevers et al., 2013; He et al., 2022). These uptake differences mean that off-target risk profile and efficacy are both species- and tissue-dependent, and cannot be extrapolated from model insects to natural-enemy species without empirical validation.

## 20 Food safety and edible tissue residues

CNTs can penetrate the seed coat and enter root systems through either cell-wall pores or by generating new pores at the seed–water interface during imbibition (Rico et al., 2011). In tomato, MWCNTs added to hydroponic systems are taken up into roots and translocated into fruits, where they measurably alter the fruit metabolome as quantified by LC-MS (McGehee et al., 2017). MWCNTs have been detected inside root cells of mature *Brassica juncea* (mustard) using TEM and confocal Raman spectroscopy (Chen et al., 2015). While direct translocation from root to harvested leaves or fruits has been demonstrated in several crops, the absolute mass of CNTs reaching edible tissues is typically very low-often below µg kg⁻¹ fresh-weight thresholds.

A critical food-safety concern is that CNTs can act as carriers for hydrophobic organic contaminants, increasing or decreasing their bioavailability depending on species, soil type, and CNT concentration. MWCNTs co-applied with weathered chlordane or DDx decreased pesticide uptake by 21–80% across zucchini, corn, tomato, and soybean- generally a beneficial effect (De La Torre-Roche et al., 2013). By contrast, C_60_ fullerenes increased chlordane uptake by up to 34.9% in certain species, illustrating that the chemical nature of the nanocarbon matters significantly (De La Torre-Roche et al., 2013). For BioCNT-TLS, the practical implication is that regulatory evaluation should not only consider the CNT itself but also its effect on the bioavailability of any co-deployed agrochemical.

## 21 Environmental fate and the green nanomaterial paradigm

**Figure 6:**
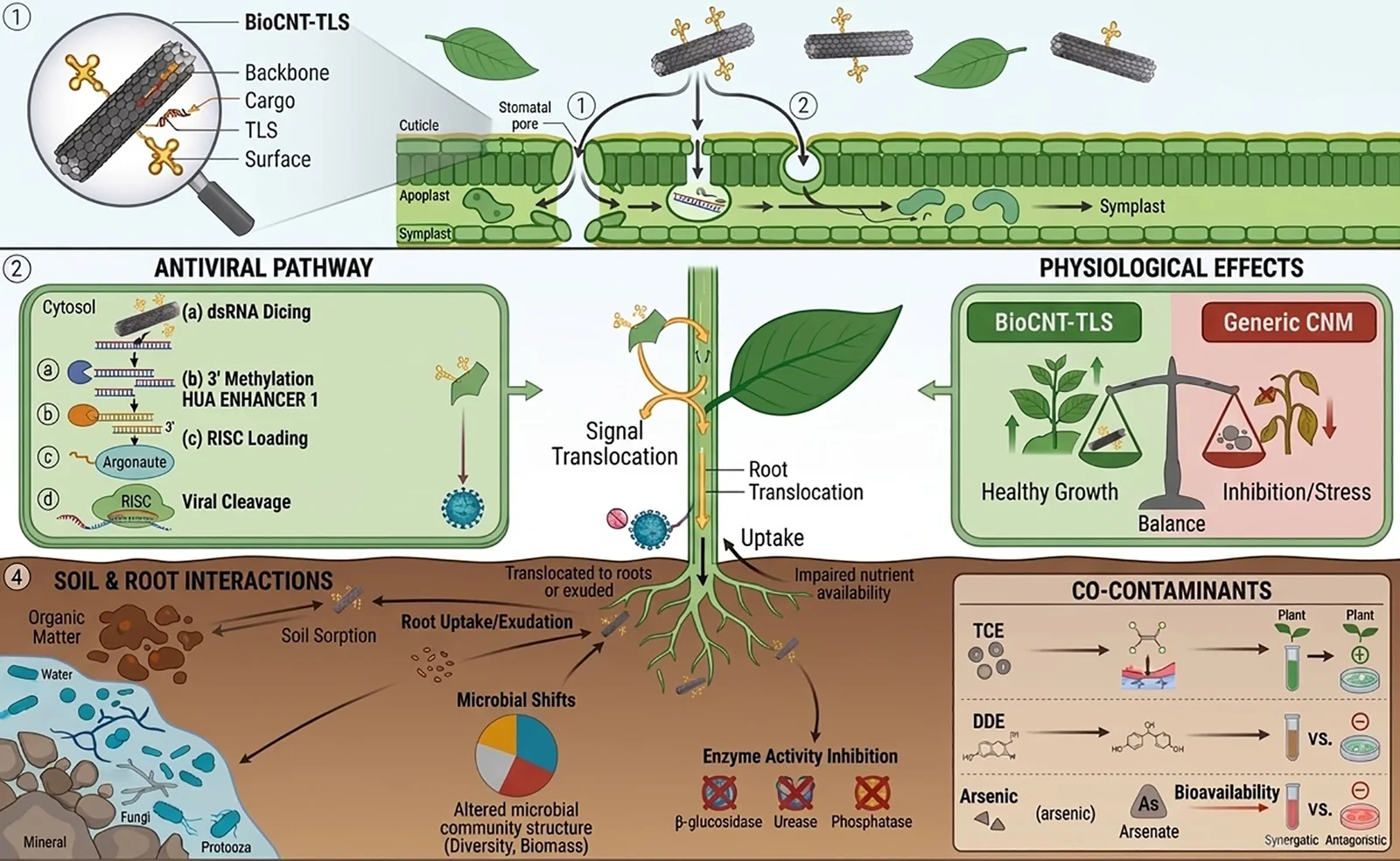
The BioCNT-TLS engineered mobile resistome: from systemic antiviral silencing to field-scale environmental fate.Figure depicts the structural composition of the BioCNT-TLS nanocarrier, comprising a functionalized single-walled carbon nanotube backbone, dsRNA/shRNA cargo, tRNA-like structure motifs for vascular translocation, and tailored surface chemistry, which together enable cellular internalization via stomatal pores and apoplastic/symplastic routes. Intracellular antiviral pathway: dsRNA is diced into 21–24 nt siRNAs by Dicer-like enzymes, 3’-methylated by HEN1 to prevent degradation, loaded into Argonaute within the RNA-induced silencing complex, and used for sequence-specific cleavage of invading viral genomes, with TLS motifs driving phloem-mediated translocation and systemic protection of distal tissues. Physiological effects of BioCNT-TLS against generic carbon nanomaterials, illustrating the targeted antiviral efficacy and minimized phytotoxicity of properly engineered formulations relative to non-engineered or improperly calibrated controls. Soil and root interactions panel outlines the post-application environmental interactions of residual BioCNTs, including soil sorption with organic matter and minerals, shifts in soil microbial community composition and biomass, inhibition of key soil enzymes (β-glucosidase, urease, phosphatase), and modulation of co-contaminant bioavailability (trichloroethylene, dichlorodiphenyldichloroethylene, arsenic), which may yield synergistic or antagonistic effects on plant health depending on the chemical identity of the nanocarbon and the co-formulated agrochemical.

The deployment of BioCNTs at the field scale necessitates a comprehensive understanding of their environmental lifecycle, from initial application to final degradation (Mukherjee et al., 2016; Zaytseva and Neumann, 2016). SWCNTs are known for their high chemical stability, which is advantageous for cargo protection but raises concerns about their accumulation in the rhizosphere and potential for bio-magnification (Kah et al., 2021; Kumari et al., 2023). Research has shown that functionalized nanotubes can interact with soil organic matter, potentially altering the bioavailability of other agrochemicals or influencing the composition of soil microbial communities (**Figure 6**) (Mukherjee et al., 2016; An et al., 2022). However, the use of biodegradable surface coatings, such as chitosan or low-molecular-weight polymers, can facilitate the eventual breakdown of the delivery complex, mitigating long-term ecological risks (An et al., 2022; Balusamy et al., 2023). It is imperative to develop ecologically-informed nanobionics that utilize natural degradation triggers such as soil-dwelling enzymes or UV exposure, to ensure that the delivery vehicle does not persist beyond its functional window.

### 21.1 Occupational exposure and regulatory framework gaps

Quantitative occupational exposure data for spray operators handling BioCNT-TLS formulations during mixing, loading, and spray application are not yet available in the peer-reviewed literature. Conventional agrochemical exposure protocols do not capture nano-specific inhalation and dermal exposure routes generated by dry-powder fragmentation during dosing, by spray-suspension aerosolisation, or by re-entry intervals for freshly treated foliage. Kah et al. (2021) proposed a comprehensive human-health risk-assessment framework specifically for nanopesticides; importantly, that framework has not yet been formally adopted by regulatory agencies, and BioCNT-TLS formulations have not been registered under any nanopesticide-specific pathway. From a regulatory standpoint, foliar-applied dsRNA products already have a precedent. Fletcher et al. (2020) reviewed the EPA FIFRA Section 3 registration of Ledprona as a regulatory pathway for RNAi-based biopesticides, and Dietz-Pfeilstetter et al. (2021) documented parallel US and EU regulatory considerations for dsRNA-based externally applied pesticides. The addition of a BioCNT carrier component adds a second regulatory layer requiring nano-specific safety evaluation of persistence, co-contaminant-vector behaviour, and nano-form-specific exposure (Kah et al., 2021; Kumari et al., 2023). Additionally, it was noted that harmonised international guidance for SIGS products remained pending (Wang and Jin, 2017), and the BioCNT-TLS system inherits that gap from both its RNAi and nanocarbon components (Dietz-Pfeilstetter et al., 2021; Kumari et al., 2023).

## 22 Cross-kingdom RNAi: variability, resistance, and off-target risk management

Insect and fungal sensitivity to exogenous dsRNA varies by orders of magnitude across taxa. Coleoptera and Hemiptera in the suborder Auchenorrhyncha show consistently strong RNAi responses, often at nanomolar dsRNA doses, while Lepidoptera, Orthoptera, and many Homoptera require substantially higher doses (Nitnavare et al., 2021; He et al., 2022). Diptera lack orthologues of the SID-1 dsRNA-channel protein and instead use clathrin-dependent endocytosis for dsRNA uptake-a mechanism shared with *Caenorhabditis elegans* (Saleh et al., 2006; Shih and Hunter, 2011; Swevers et al., 2013). In some species, both SID-1 and SID-2 pathways operate, while in others, neither has been experimentally confirmed (Nitnavare et al., 2021; He et al., 2022). These differences affect dose calculations for BioCNT-mediated delivery, and the formulation will need to be tuned to the most RNAi-sensitive species in the targeted pest complex.

Several insect species have shown reduced RNAi sensitivity after prolonged selection pressure, mediated by changes in dsRNase expression, RISC suppression, and altered gut-uptake mechanisms. Although such resistance is not yet widespread in the field, the evolutionary risk is real and should drive design choices e.g., multi-target dsRNA pyramids targeting conserved essential genes, as demonstrated in aphid management work (Fletcher et al., 2020; Nitnavare et al., 2021). Avoiding targeting essential genes with high copy number variation or known propensity for compensatory paralogue expression is an additional safeguard.

A pre-deployment risk evaluation for any BioCNT-TLS formulation should include: (a) taxon-specific RNAi sensitivity testing in a panel of target pests and at least three non-target species (one pollinator, one predator, one parasitoid); (b) bioinformatic off-target screening using at minimum dsCheck + si-Fi + a custom BLAST against locally relevant transcript databases (Naito et al., 2005; Horn and Boutros, 2010; Lück et al., 2019); (c) explicit verification that no ≥21-nt match exists against any insect, bee, or endangered pollinator transcript above the experimentally validated threshold; (d) integration of multi-target designs to slow resistance evolution. These procedures mirror the precautionary framework described by EFSA and the US EPA for dsRNA-based biopesticides (Fletcher et al., 2020; Romeis and Widmer, 2020; Dietz-Pfeilstetter et al., 2021; Nitnavare et al., 2021; Rank and Koch, 2021; He et al., 2022); (e) standardised acute, chronic, and reproductive toxicity assessment for at least two non-Apis pollinator species exposed orally, dermally, and by contact to the full BioCNT-TLS formulation, recognising that spray-operator exposure routes are not captured by conventional agrochemical protocols but fall within the human-health risk-assessment framework for nanopesticides (Kah et al., 2021); and (f) chronic low-dose ecotoxicity testing over at least two cropping cycles conducted at environmentally relevant predicted environmental concentrations (ng-µg kg⁻¹, rather than at the 10-10,000 mg kg⁻¹ range typical of most current CNT ecotoxicology studies (Liné et al., 2017; Adeel et al., 2021b).

## 23 Socio-economic and regulatory barriers: navigating the GMO landscape

The commercial trajectory of BioCNT-TLS platforms is inextricably linked to the evolving regulatory definitions of genetic modification. Currently, the international community is divided on whether spray-induced gene silencing products should be classified as conventional biopesticides or as temporary genetically modified organisms (Kumari et al., 2023). Unlike traditional transgenic crops, which contain permanent DNA insertions, spray-induced gene silencing (SIGS) utilizes transient RNA molecules that do not integrate into the host genome (Cagliari et al., 2019; Mezzetti et al., 2020). However, the use of carbon nanotubes as a delivery vehicle introduces a secondary layer of scrutiny regarding the environmental persistence and safety of nanomaterials in the food chain (An et al., 2022). Proponents argue that the lack of heritability in SIGS should facilitate a more streamlined approval process similar to that of biochemical pesticides, potentially reducing the time-to-market from decades to years (Dietz-Pfeilstetter et al., 2021; Rank and Koch, 2021). The regulatory limbo surrounding SIGS represents a significant bottleneck for private sector investment. One must advocate for a bifurcated regulatory framework that separates the safety assessment of the inert nanocarrier from the sequence-specific risk of the RNA payload, thereby allowing for faster adaptation to emerging viral threats.

From an economic perspective, the high cost of high-purity dsRNA and the large-scale synthesis of functionalized SWCNTs remain the primary hurdles to broad-acre adoption (Dubrovina and Kiselev, 2019; Rank and Koch, 2021). While the nanoprimer strategy can enhance the bioavailability of the RNA cargo, the sheer volume of material required for foliar application in crops like maize or wheat necessitates significant advancements in cell-free RNA production and low-cost nanotube purification (Dubrovina and Kiselev, 2019; Balusamy et al., 2023). Furthermore, the socio-economic impact on smallholder farmers must be considered; if the technology remains proprietary and expensive, it could exacerbate the digital and molecular divide in global agriculture (Mukherjee et al., 2016; Kumari et al., 2023). Decentralized, bio-based production models for both the nanocarriers and the RNA effectors may provide a pathway toward more equitable access (Jiang et al., 2021; An et al., 2022). The economic viability of nanobionics is a function of scale and precision application. Moving from blanket foliar sprays to targeted drone-assisted applications could reduce material costs by an order of magnitude, making the mobile resistome accessible to a broader range of agricultural systems.

For the BioCNT-TLS platform to achieve commercial viability, the cost of the RNA payload and the nanocarrier must be drastically reduced (Dubrovina and Kiselev, 2019; Rank and Koch, 2021). Traditional fermentation-based RNA production is limited by low yields and the presence of host-cell contaminants (Dubrovina and Kiselev, 2019; Mezzetti et al., 2020). Emerging cell-free synthesis platforms utilize purified enzymes to print custom dsRNA and shRNA sequences with 100% fidelity and zero biological contamination. These systems allow for the rapid synthesis of multi-target pyramids at a fraction of the cost, bringing the price of the mobile resistome closer to parity with high-end chemical fungicides (Balusamy et al., 2023; Chen et al., 2025). The industrialization of cell-free RNA synthesis is the prerequisite for the global adoption of nanobionics. Researchers must focus on developing decentralized bioprocessing units that can be deployed at the regional level, allowing farmers to print custom antiviral sequences tailored to the local viral quasispecies.

## 24 Precision breeding: integrating nanobionics with data-driven agriculture

The future of crop immunity lies in the integration of BioCNT-TLS platforms with the Internet of Things and satellite-based disease monitoring (Jiang et al., 2021; Balusamy et al., 2023). The proactive intervention would leverage the systemic mobility of the TLS motif to protect not only the infected plants but also a buffer zone of healthy neighbors, effectively creating a genomic firewall (Biedenkopf et al., 2020; Liu et al., 2026). This synergy between molecular biology and precision engineering represents the next evolution of integrated pest management (Wang and Jin, 2017; Xi et al., 2025). Critically, the genomic firewall concept shifts the focus from treating symptoms to programming landscape-level resilience. This approach requires a substantial rethinking of agricultural data sharing, as the efficacy of the molecular response depends on the speed and accuracy of the detection network.

Moreover, the spray-to-edit capability of BioCNTs could allow for real-time epigenetic modulation in response to abiotic stressors such as heat or drought (Ahmar et al., 2021; Nadakuduti and Enciso-Rodríguez, 2021). By delivering small RNAs that target DNA methyltransferases or histone modifiers, farmers could tune the plant’s stress-response genes during a single growing season without the need for permanent genetic changes (Dubrovina and Kiselev, 2019; Pandey et al., 2019). This flexibility is essential for adapting to the unpredictable climate patterns of the 21^st^ century, providing a level of resilience that traditional breeding cannot match (Daniel et al., 2024).

## 25 Outlook: integration with digital and precision agriculture

Economic modeling indicates that the precision application of BioCNT-TLS SIGS using autonomous drones or AI-guided sprayers can reduce the total material required per hectare compared to blanket chemical applications (Rank and Koch, 2021; Ghosh et al., 2023). Targeted foliar application via precision spraying platforms may reduce the total nanomaterial load per hectare compared with blanket sprays, although quantitative field-scale cost analyses are not yet available. (Biedenkopf et al., 2020; Liu et al., 2026). This shift toward data-driven molecular agriculture is essential for ensuring that high-performance biotechnology is accessible to both large-scale commercial operations and smallholder farmers (Mukherjee et al., 2016; Kumari et al., 2023). Empirically, the economic viability of the mobile resistome is inextricably linked to the intelligence of the application system. Without the integration of real-time disease detection and variable-rate application technology, the high cost of advanced nanobionics may limit its use to only the most profitable horticultural crops, thereby widening the gap between different agricultural sectors. In principle, combining early-stage remote disease detection with targeted BioCNT-TLS application would represent a closed-loop crop health management system, but no published system currently integrates all of these components. Realizing such integration will require coordinated advances in (i) hyperspectral disease signatures, (ii) field robotics, and (iii) nanoformulation regulation.

## 26 Conclusion

The synthesis of nanotechnology and plant vascular biology in the BioCNT-TLS platform marks the beginning of a nanobionic era in sustainable agriculture. By solving the dual challenges of RNA instability and lack of systemic mobility, this technology provides a precision-targeted, non-transgenic solution to the global crisis of plant viral epidemics. The engineering of a ‘mobile resistome’ capable of navigating the vascular highway to protect distal tissues represents a fundamental shift from reactive pesticide use to systemic, RNA-mediated pre-emptive protection. While significant technical and regulatory hurdles remain, the prospect of programming whole-plant immunity from a single topical application is appealing-but only if the field-deployment safety criteria set out in Sections 13–15 are quantitatively demonstrated before any commercial registration, including pollinator chronic toxicity testing at multiple non-Apis taxa (Mommaerts et al., 2012; Heard et al., 2017; Hooven et al., 2019), bioaccumulation kinetics at environmentally relevant PEC-aligned concentrations (Flores-Cervantes et al., 2014; Liné et al., 2017; Adeel et al., 2021b), and operator-exposure risk assessment under the comprehensive nanospecific framework proposed by Kah et al. (2021). As we move forward, the focus must shift from laboratory-scale proofs-of-concept to holistic field-level assessments that integrate molecular efficacy with ecological safety. The ultimate success of this technology will depend on our ability to build a transparent, evidence-based framework that balances the urgent need for food security with the long-term health of our planet’s ecosystems. In the face of a rapidly changing climate and evolving pathogen landscape, the BioCNT–TLS platform offers a robust and flexible toolkit for the development of RNA-mediated crop protection strategies.

## 27 Box 1. Regulatory readiness checklist

Prior to any field deployment of BioCNT-TLS, the following data gaps should be filled: (i) experimentally validated chronic (≥2 cropping seasons) soil accumulation data at environmentally relevant ng–µg kg⁻¹ concentrations rather than at the 10-10,000 mg kg⁻¹ range used in most current CNT ecotoxicology tests (Liné et al., 2017; Adeel et al., 2021b); (ii) acute, chronic, and reproductive toxicity data for at least two non-Apis pollinator species exposed to the BioCNT-TLS formulation, drawing on the existing silica-nanoparticle *Bombyx terrestris* reproductive-failure model (Mommaerts et al., 2012) and on the comparative-bee-taxa framework as proposed earlier (Heard et al., 2017); (iii) mixture-toxicity characterisation with the agrochemicals most likely to be co-applied, recognising that carbon nanotubes can either reduce (MWCNTs reducing chlordane and DDx uptake by 21-80% across zucchini, corn, tomato, and soybean (De La Torre-Roche et al., 2013);) or increase (C_60_ fullerenes increasing uptake of certain metals (Leroy et al., 2022);) the bioavailability of co-contaminants depending on the chemical identity of the nanocarbon; (iv) occupational exposure characterisation during mixing, loading, and spray application following the comprehensive nano-specific risk-assessment framework proposed by Kah et al. (2021); and (v) a life-cycle-assessment model that captures both the short-lived RNA payload (hours to days) and the long-lived BioCNT carrier (years to decades) components of the formulation (Flores-Cervantes et al., 2014). Until these data are obtained, BioCNT-TLS should be regarded as a research-grade delivery route rather than a deployable agronomic practice.

## 28 Outstanding questions

- What is the long-term fate and potential accumulation of carbon nanotubes in plant tissues and the surrounding soil ecosystem after multiple growing seasons?
- How do variable field conditions, such as significant changes in temperature or water availability, affect the speed and efficiency of the plant’s vascular transport for these delivery complexes?
- Does the introduction of synthetic RNA trafficking motifs compete with or disrupt the plant’s natural internal communication and developmental signaling?
- What is the minimum concentration of RNA that must be successfully delivered to a single leaf to ensure a robust and systemic immune response throughout the entire plant?
- Can viral populations evolve to evade the delivered RNA sequences by mutating their replication genes, and how frequently would the treatment need to be updated?
- What are the specific biochemical triggers within the cytoplasm that cause the RNA to detach from the carbon nanotube surface to begin the silencing process?
- To what extent can the systemic RNA signals be transferred to non-target organisms, such as soil microbes or pollinating insects, through cross-kingdom pathways?
- Does the immunity induced by these topical applications persist in any form through the seeds to provide protection for the next generation of crops?
- What are the primary technical and economic barriers to producing high-purity RNA and functionalized nanotubes at the scale required for broad-acre farming?
